# Ellagitannin Oligomers from *Eucalyptus camaldulensis* Leaves and Their Role in the Detoxification of Aluminum

**DOI:** 10.3390/molecules30102216

**Published:** 2025-05-19

**Authors:** Haruna Uemori, Ayano Inoue, Shoichi Suzuki, Yuji Iwaoka, Tsutomu Hatano, Morio Yoshimura, Yoshiaki Amakura, Toshiyuki Murakami, Ko Tahara, Hideyuki Ito

**Affiliations:** 1 Doctorate Course of Health and Welfare Science, Graduate School of Okayama Prefectural University, 111 Kuboki, Soja 719-1197, Okayama, Japan; h-uemori@maruzenpcy.co.jp (H.U.); iwaoka@fhw.oka-pu.ac.jp (Y.I.); 2 Research Center, Maruzen Pharmaceuticals, Co., Ltd., 1089-8 Sagata, Shinnichi-Cho, Fukuyama 729-3102, Hiroshima, Japan; to-murakami@maruzenpcy.co.jp; 3 Faculty of Health and Welfare Science, Okayama Prefectural University, 111 Kuboki, Soja 719-1197, Okayama, Japan; ayano-.-10251@outlook.jp (A.I.); ssho5532@gmail.com (S.S.); 4 Division of Pharmaceutical Sciences, Graduate School of Medicine, Dentistry, Pharmaceutical Sciences, Okayama University, 1-1-1 Tsushima-Naka, Kita-ku 700-8530, Okayama, Japan; hatano-t@cc.okayama-u.ac.jp; 5 Pharmaceutical Sciences, Matsuyama University, 4-2 Bunkyo-cho, Matsuyama 790-8578, Ehime, Japan; myoshimu@g.matsuyama-u.ac.jp (M.Y.); amakura@g.matsuyama-u.ac.jp (Y.A.); 6 Forestry and Forest Products Research Institute, 1 Matunosato, Tsukuba 305-8687, Ibaraki, Japan; taharako@affrc.go.jp; 7 National Institutes of Biomedical Innovation, Health and Nutrition, Kento Innovation Park, NK Building, 3-17 Senrioka, Shinmachi 566-0002, Osaka, Japan

**Keywords:** *Eucalyptus camaldulensis* Dehnh., ellagitannin oligomer, eucamalin A, eucarpanin D_2_, eurobustin C, Al detoxification

## Abstract

*Eucalyptus camaldulensis* of the Myrtaceae family shows high resistance to aluminum (Al) ions and contains various compounds such as steroids, terpenoids, saponins, flavonoids, glycosides, alkaloids, and tannins. Although the ellagitannin oenothein B (**12**) isolated from *E. camaldulensis* exhibits remarkable properties for Al detoxification, likely contributing to its Al resistance, other ellagitannin oligomers present in *E. camaldulensis* have not been investigated in detail. In this study, novel dimeric and trimeric ellagitannin oligomers eucarpanin D_2_ (**1**) and eucamalin A (**2**), together with known gallotannins (**7**, **8**, and **10**), monomeric ellagitannins (**4**–**6**, and **11**), and dimeric ellagitannins (**3**, **9**, and **12**–**14**), were isolated from *E. camaldulensis* leaves. The structures of these novel compounds were elucidated based on their chemical and physicochemical properties, including the orientations of tergalloyl groups in compounds **1** and **2**. Similar to compound **12**, previously isolated from the roots of *E. camaldulensis*, the ellagitannins demonstrated good Al detoxification properties. Hence, these tannins may play a critical role in the high Al resistance of *E. camaldulensis* in acidic soils. This paper reports for the first time the isolation of ellagitannin oligomers from the leaves of *E. camaldulensis*.

## 1. Introduction

*Eucalyptus camaldulensis* Dehnh. is an evergreen tree that belongs to the Myrtaceae family. Native to Australia, it is widespread in tropical and subtropical regions and commonly grows on riverbanks. This plant is tolerant to extreme drought and high temperatures, grows rapidly, and exhibits a good coppicing ability [1,2]. Therefore, it shows significant variations among different regions. *E. camaldulensis* exhibits high resistance to Al ions and can grow in acidic soils, which account for 30% of the Earth land area [3,4]. We conducted an ingredient search to elucidate the mechanism of this ability of *E. camaldulensis*.

*E. camaldulensis* contains various compounds such as steroids, terpenoids, saponins, flavonoids, glycosides, alkaloids, and tannins [5,6]. Among them, we previously identified the Al-binding ligand oenothein B (**12**), a dimeric ellagitannin, in the roots of *E. camaldulensis*. It contains large amounts of compound **12**, and the bioassays using Arabidopsis (*Arabidopsis thaliana*) showed that the inhibition of root elongation by Al was alleviated by compound **12** [7]. That is, it is suggested that compound **12** contributes to the Al detoxification in *E. camaldulensis*. Initially, compound **12** was isolated from the leaves of *Oenothera erythrosepala* (Onagraceae), which is widely distributed among species belonging to the Myrtaceae, Onagraceae, and Lythraceae families [8]. These species have oligomeric analogs, such as eucarpanin D_1_ (**15**) (dimer), oenothein T_1_ (trimer), eucarpanin T_1_ (**16**), and T_2_ (trimer) extracted from the leaves of *Eucalyptus cypellocarpa* and woodfordin I (dimer), E (trimer), and F (tetramer) isolated from the flowers of *Woodfordia fruticosa* [8,9,10]. However, the ellagitannin oligomers present in *E. camaldulensis* have not been investigated in detail to date. Therefore, we focused on high molecular weight tannins to identify those having higher ability to reduce the toxic effects of Al than compound **12**, as well as the leaves of *E. camaldulensis*, which are rich in such tannins. In this study, we isolated and characterized monomeric and oligomeric ellagitannin, along with gallotannins, from the leaves of *E. camaldulensis* and examined their Al detoxification properties.

## 2. Results and Discussion

### 2.1. Isolation and Structural Elucidation of Novel and Known Compounds

**Figure 1:**
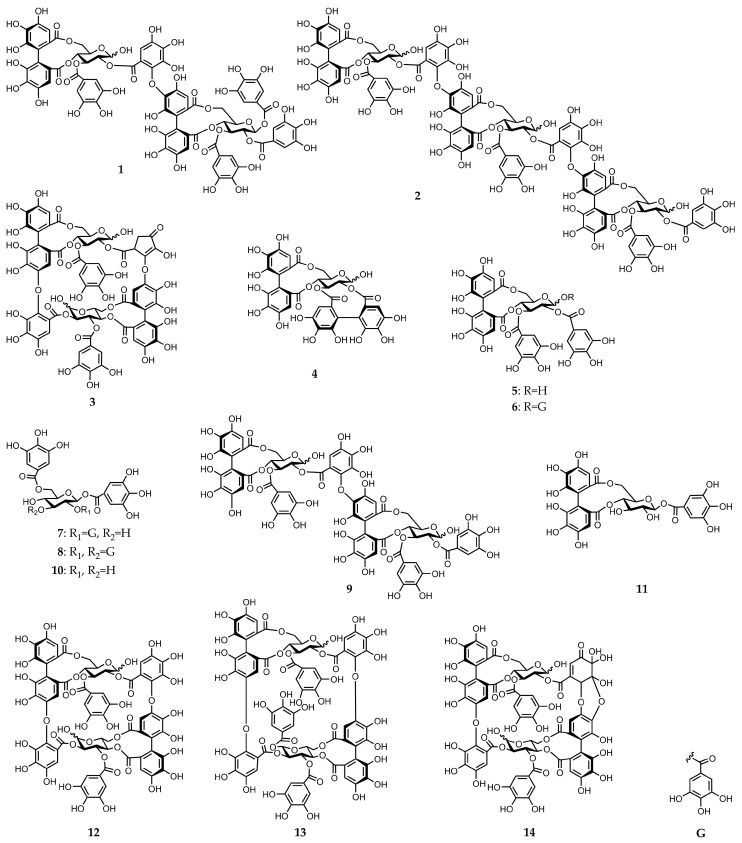
Chemical structures of gallotannins and ellagitannins isolated from *E. camaldulensis* leaves (compounds **1**–**14**).

A concentrated solution of a 70% aqueous acetone homogenate from the frozen leaves of *E. camaldulensis* was sequentially extracted with Et_2_O, EtOAc, and water-saturated *n*-BuOH. The EtOAc extract yielded three known ellagitannins: pedunculagin (**4**) [11], tellimagrandin I (**5**) [12], and tellimagrandin II (**6**) [12], two known gallotannins: 1,2,6-tri-*O*-galloyl-*β*-d-glucose (**7**) [13] and 1,2,3,6-tetra-*O*-galloyl-*β*-d-glucose (**8**) [14], and dimeric ellagitannin: eucalbanin C (**9**) [15]. In addition, two novel dimeric and trimeric ellagitannins: eucarpanin D_2_ (**1**) and eucamalin A (**2**), were isolated from this extract (Figure 1). The *n*-BuOH extract gave gallotannin: 1,6-di-*O*-galloyl-*β*-d-glucose (**10**) [16], three monomeric ellagitannins: compounds **4** and **5**, and strictinin (**11**) [11,17], two dimeric ellagitannins: compound **9** and oenothein B (**12**) [8,18], and two highly oxidized ellagitannins: eurobustin C (**3**) [19] and eugeniflorin D_2_ (**14**) [8,18,19] (Figure 1). The water-soluble extract obtained dimeric ellagitannin: eugeniflorin D_1_ (**13**) [8,19] (Figure 1). The known compounds were identified by comparing their spectroscopic properties to data obtained from previous isolations (Figure S1).

We further characterized the novel dimeric and trimeric ellagitannins identified in this work and named them eucarpanin D_2_ (**1**) and eucamalin A (**2**), respectively. These ellagitannins contained tergalloyl groups serving as acyl units. Eurobustin C (**3**) was first isolated from *Eucalyptus robusta*, and its structure was identified by Yoshida et al. in their book, however, a detailed chemical structure determination was not reported [19]. Therefore, we performed a detailed structural characterization of compound **3**. The chemical structures of these ellagitannins are described below.

**Figure 2:**
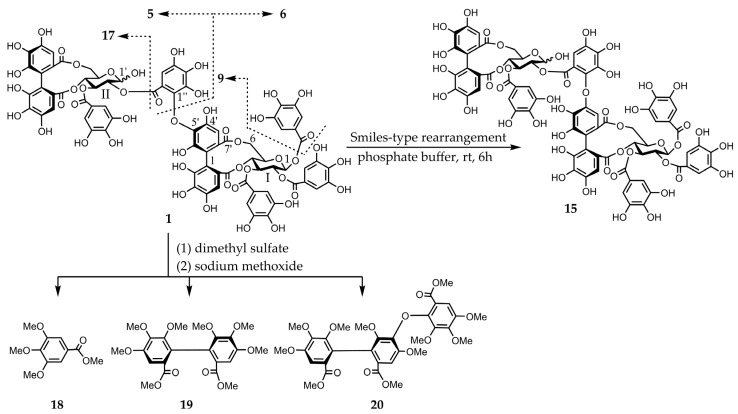
Smiles-type rearrangement, partial hydrolysis, and methylation of compound **1** followed by methanolysis.

Eucarpanin D_2_ (**1**) was obtained as a pale-brown amorphous powder. The molecular formula of compound **1** (C_75_H_54_O_48_) was determined by high-resolution electrospray ionization mass spectrometry (HR-ESI-MS) from its singly and doubly charged molecular ions at *m/z* 1721.1649 [M − H]^−^ and 860.0840 [M − 2H]^2−^, indicating that compound **1** was an ellagitannin dimer. Its ^1^H-nuclear magnetic resonance (NMR) spectrum is similar to that of eucalbanin C (**9**) and indicated the presence of four sets of 2H singlets (δ 7.11, 7.11, 7.02, 7.02, 7.01, 7.01, 7.00, and 6.99), two sets of 1H singlets (δ 6.65, 6.64, 6.49, and 6.48), and three sets of 1H singlets (δ 6.87, 6.84, 6.60, 6.59, 6.53, and 6.51), which were ascribed to four galloyl, one hexahydroxy diphenoyl (HHDP), and one tergalloyl groups, respectively. This compound also exists as an equilibrium mixture of two tautomers and is characterized by the presence of two ^4^C_1_-conformation glucopyranose cores. The absolute configuration of glucopyranose in compound **1** was determined from the D-series using the method described by Tanaka et al. [20]. The H-1 signal of the *β*-anomer of compound **1** shifted to a lower field (δ 6.18, d, *J* = 8.4 Hz) than that of compound **9** (δ 4.98). The corresponding ^13^C-NMR spectrum contains a signal characteristic of the tergalloyl group in the hydroxyl carbon region; the tergalloyl-4’ carbon was present in a lower field (δ 149.0) than that of the valoneoyl group, similar to that of compound **9** (Figure S2). The enzymatic hydrolysis of compound **1** with tannase yielded compound **9**. Tellimagrandin I (**5**), tellimagrandin II (**6**), and gemin D (**17**) [21] were produced via partial hydrolysis. This isomerization via a Smiletype rearrangement was successfully applied to establish a chemical correlation between compound **1** and eucarpanin D_1_ (**15**). The tergalloyl group in the crowded substitution mode is isomerized to the valoneoyl group, which has a less sterically hindered structure because of a Smiles-type rearrangement. An aqueous solution containing a small amount of 0.02 M phosphate buffer (pH 7.4) was left standing at room temperature for 6 h to produce an isomerized product of compound **15**. The methylation of compound **1** with dimethyl sulfate and potassium carbonate in acetone followed by methanolysis yielded methyl tri-*O*-methylgallate (**18**), dimethyl hexamethoxydiphenate (**19**), and trimethyl octa-*O*-methyltergallate (**20**) (Figure 2). The positive cotton effect in the short wavelength region of the circular dichroism (CD) spectrum indicated that the absolute configuration of HHDP and tergalloyl group was *S*-series, respectively [22]. Based on these data, eucarpanin D_2_ is represented by the structure of compound **1**.

**Figure 3:**
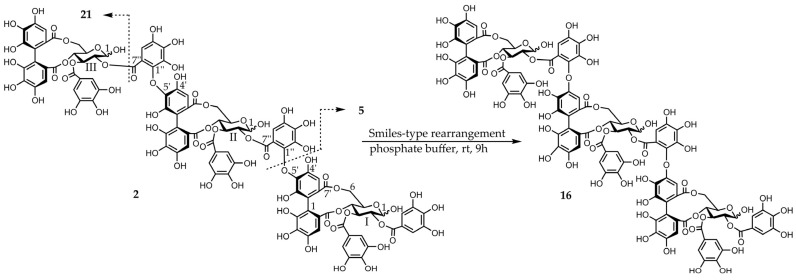
Smiles-type rearrangement and partial hydrolysis of compound **2**.

Eucamalin A (**2**) was obtained as a pale-brown amorphous powder. The molecular formula of this material, which was determined by HR-ESI-MS from the singly, doubly, and triply charged molecular ions at *m*/*z* 2353.2400 [M − H]^−^, 1176.1117 [M − 2H]^2−^, and 783.7383 [M − 3H]^3−^, respectively, was C_102_H_74_O_66_, indicating that compound **2** was a trimeric ellagitannin. The ^1^H-NMR spectrum pattern of compound **2** was strongly resembled those of compound **9** and eucarpanin T_1_ (**16**) [23], suggesting that compound **2** was linked through its tergalloyl groups. Although the ^1^H-NMR spectrum of compound **2** exhibited intricate signals caused by the presence of anomeric mixtures at the glucose cores, the presence of galloyl, HHDP, tergalloyl groups, and glucose moieties was confirmed by the signals at δ 7.06–6.96 (galloyl-H), δ 6.87–6.83 (tergalloyl-H), δ 6.64–6.62 (HHDP-H), δ 6.60–6.56 (tergalloyl-H), δ 6.52–6.46 (tergalloyl and HHDP-H), and δ 3.74–5.90 (glucose-H) (Figure S3). Furthermore, the signal pattern of glucose carbon atoms in the ^13^C-NMR spectrum of compound **2** was similar to those of compounds **9** and **16**. The absolute configuration of glucopyranose in compound **2** was identified from the D-series [20]. This isomerization via a Smiles-type rearrangement was successfully applied to establish a chemical correlation between compounds **2** and **16**. An aqueous solution of compound **2** containing a small amount of the 0.02 M phosphate buffer (pH 7.4) was left to stand at room temperature for 6 h and was nearly identical to those of compound **16**. The phenolic acyl units of compound **2** were confirmed by the methylation of compound **2** and subsequent methanolysis to produce compounds **18**–**20**. The configurations of the chiral biphenyl moieties of the HHDP and tergalloyl units in compound **2** represent the *S*-series because of the strong positive cotton effects in the CD spectrum at 228 and 220 nm (shoulder), respectively [22]. The partial hydrolysis of compound **2** generated compounds **5** and **17** (Figure 3). Based on these data, we determined the structure of compound **2**.

**Figure 4:**
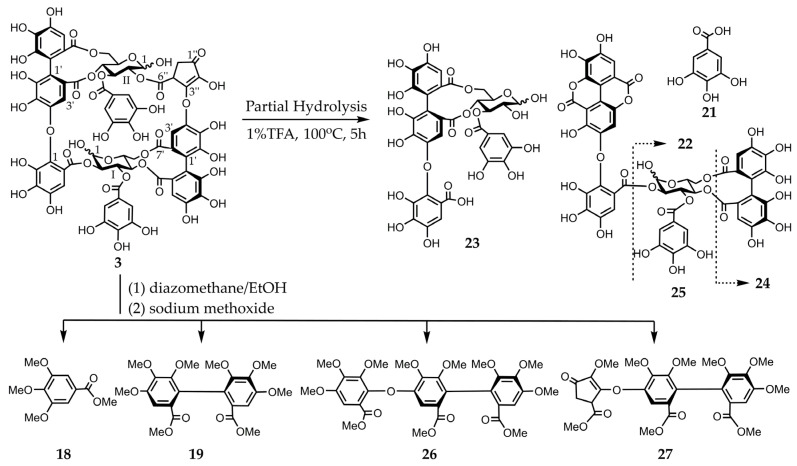
Partial hydrolysis and methylation of compound **3** followed by methanolysis.

Eurobustin C (**3**) was obtained as a pale-brown amorphous powder. The molecular formula of this material (C_67_H_48_O_43_) was determined by HR-ESI-MS from its singly and doubly charged molecular ions with *m*/*z* 1539.1491 [M − H]^−^ and 769.0657 [M − 2H]^2−^, indicating that compound **3** was an ellagitannin dimer. Some of the aromatic and sugar proton signals in the obtained ^1^H-NMR spectrum was broadened probably because of the poor flexibility of the macro-ring arising from restricted rotation. The ^1^H-NMR spectrum of compound **3** exhibited two 2H singlets at δ 7.15 and 7.08 attributable to the protons of one galloyl group. Three 1H singlets at δ 7.18, 6.85, and 6.56, and two 1H singlets at δ 6.39 and 6.26 indicated the presence of an HHDP group and a new acyl unit named a eurobustinoyl group. This unit is metabolized by the dehydrovaloneoyl group of eugeniflorin D_2_ (**14**). Therefore, the ^1^H-NMR spectrum of compound **3** is similar to that of compound **14**. ^1^H-^1^H correlation spectroscopy (COSY) experiment revealed the presence of two ^4^C_1_ conformation glucopyranose cores. The doublet at δ 6.94 (*J* = 3.6 Hz) and δ 4.84 (*J* = 8.8 Hz) of anomeric protons resonated at a higher field than those expected for the C-1 signals of acylated *α*- and *β*-anomers, demonstrating that both anomeric centers were unacylated. A methine proton signal at δ 4.45 and methylene proton signals at δ 2.14 and 1.89 were coupled to each other. The ^13^C-NMR spectrum of compound **3** showed the presence of an *α*, *β*-unsaturated ketone system (δ 136.9, 160.6, and 197.2). Heteronuclear multiple bond connectivity (HMBC) experiment revealed the existence of correlations between methyl protons and carbonyl carbon (δ 197.2), which were separated by two bonds. The ^13^C-NMR spectrum was similar to those of brevifolincarboxylic acid [19]. Therefore, this compound contains a five-membered ring according to the HMBC experiment of compound **3**. The connectivity between a methine protons (δ 4.45) and one of the methylene protons (δ 2.14), and H-2 of glucose II (δ 4.82, t, *J* = 8.8 Hz) was confirmed by a long-range correlation through the same ester carbonyl carbon signal (δ 170.5). Similarly, H-3’ of the valoneoyl group (δ 6.85) correlated with H-4 of glucose II (δ 4.82, t, *J* = 8.8 Hz) through the signal at δ 166.9. The allocations of galloyl groups at *O*-3 of glucose I and *O*-3 of glucose II were also established by the cross peaks of H-3 of glucose I (δ 5.42, t, *J* = 8.8 Hz) and H-3 of glucose II (δ 5.92, t, *J* = 6.8 Hz), respectively, formed with the ester carbonyl carbon at δ 168.2 and 166.2, which correlated with the galloyl proton signals (Figure S4). These data suggested the formation of structure **3**, which was supported by the following chemical reaction. The partial hydrolysis of compound **3** in hot water containing 1% trifluoroacetic acid (TFA) produced gallic acid (**21**), valoneic acid dilactone (**22**), oenothein C (**23**) [24], isocoriariin F (**24**) [25,26], and cornusiin B (**25**) [26,27]. The valoneoyl group of compound **3** was of the isorugosin type because this reaction yielded compounds **23**–**25**. The methylation of compound **3** with dimethyl sulfate and potassium carbonate in acetone followed by methanolysis resulted in compounds **18** and **19**, trimethyl octa-*O*-methylvalonate (**26**), and trimethyl hexa-*O*-methyleurobustinate (**27**). Compound **19** was produced by the cleavage of the ether bond of the valoneoyl group (Figure 4). The *S*-configuration was identified for both the valoneoyl and eurobustinoyl group in compound **3** by the positive cotton effects in the CD spectrum [22]. Finally, the structure of compound **3** was established based on these data.

### 2.2. Detoxification of Al

**Figure 5:**
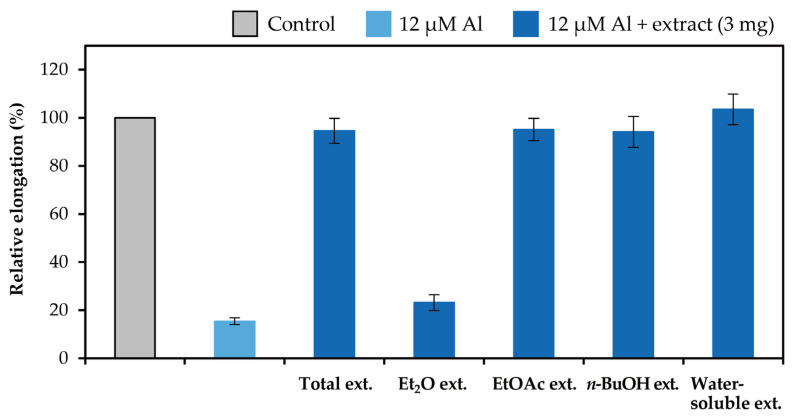
Al detoxification properties of the extracts in the liquid–liquid distributions. Roots of *A. thaliana* were exposed to 500 mL of a 2% strength modified Molecular Genetics Research Laboratory (MGRL) medium (pH 5.0) containing 0 or 12 μM AlCl_3_ and 3 mg of the extracts for 24 h. The relative root elongation is expressed as the percentage of the root elongation in the control sample (0 μM Al without extract). The values and error bars are expressed as means ± SE (*n* = 20).

**Figure 6:**
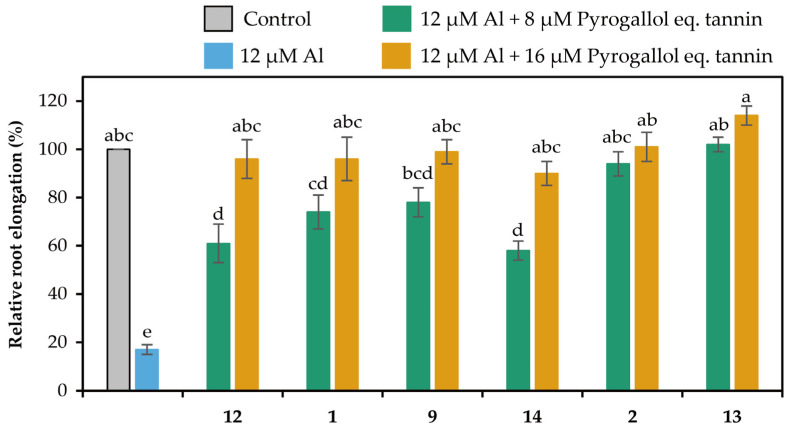
Al detoxification properties of ellagitannins (compounds **1**, **2**, **9**, **12**, **13** and **14**) isolated from *E. camaldulensis*. Roots of *A. thaliana* were exposed to a 2% strength modified MGRL medium (pH 5.0) containing 0 or 12 μM AlCl_3_ and 0, 8, or 16 μM pyrogallol-equivalent ellagitannin for 24 h. The relative root elongation is expressed as the percentage of the root elongation in the control sample (0 μM Al, 0 μM ellagitannin). The values and error bars are expressed as means ± SE (*n* = 20). The bars marked with the same letter do not differ significantly at *p* < 0.05 (Tukey-Kramer test).

**Figure 7:**
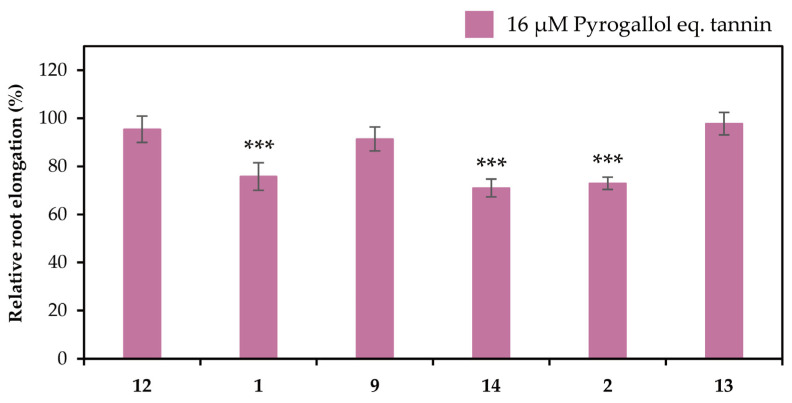
Effects of ellagitannins (compounds **1**, **2**, **9**, **12**, **13** and **14**) on the root elongation. Roots of *A. thaliana* were exposed to 0 or 16 μM pyrogallol-equivalent ellagitannin in a 2% strength modified MGRL medium (pH 5.0) for 24 h. The relative root elongation is expressed as the percentage of the root elongation in the control sample (0 μM ellagitannin). The values and error bars are expressed as means ± SE (*n* = 20). The asterisks indicate a significant difference compared with the control at *p* < 0.001 (Student’s *t*-test).

The extracts in the liquid–liquid distributions and ellagitannins isolated from leaves of *E. camaldulensis* were added to the seedlings of *Arabidopsis thaliana*, and their Al detoxification properties were evaluated after 24 h. The EtOAc, *n*-BuOH, and water-soluble extracts in the liquid–liquid distributions demonstrated Al detoxification abilities (Figure 5). Therefore, we focused on isolating tannins from these extracts. Among the ellagitannins isolated from these extracts, oenothein B (**12**), eucarpanin D_2_ (**1**), eucalbanin C (**9**), and eugeniflorin D_2_ (**14**) exhibited the same degree of root elongation, whereas eucamalin A (**2**) and eugeniflorin D_1_ (**13**) at 8 μM pyrogallol-equivalent differed significantly from compound **12** in the Tukey-Kramer test (Figure 6). We also evaluated the effect of the addition of 16 μM pyrogallol-equivalent ellagitannin alone, to the seedlings of *A. thaliana*. Among these ellagitannins: compounds **9**, **12**, and **13** did not inhibit root elongation; however, compounds **1**, **2**, and **14** slightly inhibited the root elongation process (Figure 7). These results revealed that ellagitannin did not promote root elongation and that compounds **1**, **9**, and **14** possessed the same Al detoxification ability as that of compound **12**, whereas the detoxification abilities of compounds **2** and **13** were stronger than that of compound **12**. Ellagitannins have structure of pyrogallol, and metal ions form chelate there [28,29]. Compound **12** has eight pyrogallols, which are presumed to contribute to Al detoxification by binding to Al ions [7]. Similarly, ellagitannins isolated from the leaves of *E. camaldulensis* (compounds **1**, **2**, **9**, **13** and **14**) have some or greater number of pyrogallols in their molecules as compound **12**. Further isolation of the compounds is necessary to elucidate the detailed mechanisms of Al detoxification potential, including differences in the number of pyrogallol and the steric structure of the compounds.

## 3. Materials and Methods

### 3.1. Instrumentation

Optical rotations were recorded using a Jasco DIP-1000 polarimeter (Jasco, Tokyo, Japan). The ultraviolet (UV) and CD spectra were obtained using a Jasco V-530 spectrophotometer (Jasco, Tokyo, Japan) and a Jasco J-710 spectropolarimeter (Jasco, Tokyo, Japan), respectively. The ^1^H-NMR (600 MHz) and ^13^C-NMR (151 MHz) spectra including ^1^H-^1^H COSY, heteronuclear single quantum correlation (HSQC), and HMBC were recorded on a Varian NMR instrument (Varian, Palo Alto, CA, USA), and the chemical shifts are listed in part-per-million (ppm) values relative to acetone-*d*_6_ (2.04 ppm for ^1^H and 29.8 ppm for ^13^C). The ^1^H-NMR (400 MHz) and ^13^C-NMR (100 MHz) spectra including ^1^H-^1^H COSY, HSQC, and HMBC were recorded on a JNM-ECS400 instrument (JEOL, Akishima, Tokyo, Japan), and the chemical shifts are provided in part-per-million (ppm) values relative to acetone-*d*_6_ (2.04 ppm for ^1^H and 29.8 ppm for ^13^C). The high-resolution mass spectra were obtained using a Bruker MicrOTOF II instrument (Bruker, Billerica, MA, USA) and a Xevo G3 QTof (Waters, Milford, MA, USA) with an ESI source in negative-ion mode. Reversed-phase HPLC was conducted with a Wakosil II 5C18HG (250 × 4.6 mm i.d., Wako, Osaka, Japan) column using a mobile phase composed of 0.1% aqueous TFA (solvent A) and CH_3_CN (solvent B) (82:18) and isocratic program at a temperature of 35 °C, UV detection wavelength of 250 nm, and flow rate of 0.8 mL/min (System A). Reversed-phase HPLC was performed on an Inertsustain C18 column (150 × 4.6 mm i.d., 5 µm, GL Sciences, Tokyo, Japan) using a mobile phase composed of H_2_O:CH_3_CN:HCOOH (90:5:5) (solvent A) and H_2_O:CH_3_CN:HCOOH (50:45:5) (solvent B) at 40 °C, and the gradient was programmed as follows: 0–30 min (solvent B: 0–100%) (system B). Reversed-phase HPLC was performed on a YMC-pack ODS-A column (250 × 4.6 mm i.d., 5 µm, YMC Co., Ltd., Kyoto, Japan) using a mobile phase consisting of 0.01 M H_3_PO_4_:0.01 M KH_2_PO_4_:EtOH:EtOAc = 47.5:47.5:3:2 at a column temperature of 40 °C, flow rate of 1.0 mL/min, and linear gradient. Detection was performed at 280 nm (system C). Normal-phase HPLC was conducted on a CHIRALCEL OD-H column (250 × 4.6 mm i.d., 5 µm, DAICEL Co., Ltd., Osaka, Japan) using a mobile phase composed of *n*-hexane: EtOH = 20:1 at 24 ± 1 °C. The flow rate was 2.0 mL/min, and the UV detection wavelength was 280 nm (system D). Purification by column chromatography were conducted using Diaion-HP-20 (Mitsubishi Kasei Co., Tokyo, Japan), Toyopearl HW-40 (coarse grade) (Tosoh Co., Tokyo, Japan), Sephadex LH-20 (GE Healthcare, Chicago, IL, USA), and MCI GEL CHP-20P (75–150 µm) (Mitsubishi Chemical Co., Tokyo, Japan) resins.

### 3.2. Plant Material

A clone of *Eucalyptus camaldulensis* Dehnh. (seed lot: 19708; Australian Tree Seed Center, CSIRO) was propagated by cutting as described previously. The obtained plantlets were transferred to vermiculite in plastic pots (18.4 × 11.3 cm i.d.) and cultured in a growth chamber (16 h light/8 h dark; 28/25 °C; photosynthetic photon flux density: 200 µmol m^−2^ s^−1^). The plantlets were watered twice a day with a nutrient solution [7]. After two months of cultivation, leaves were harvested from the plantlets with heights of approximately 1 m, frozen in liquid nitrogen, and stored at −80 °C until use.

### 3.3. Extraction and Isolation

The frozen leaves of *E. camaldulensis* (2.4 kg) were homogenized with 70% aqueous acetone (4.0 L × 3), filtered, and evaporated in vacuo. Extracts were obtained sequentially with Et_2_O (3.0 L × 3), EtOAc (3.0 L × 3), and water-saturated *n*-BuOH (3.0 L × 3) to yield Et_2_O (1.7 g), EtOAc (15.1 g), *n*-BuOH (40.7 g), and a water-soluble fraction (117 g). A portion of the EtOAc extract (7.0 g) was subjected to column chromatography over Toyopearl HW-40 (coarse grade) (40 × 2.2 cm i.d.) using 30%, 40%, 50%, 60%, and 70% aqueous MeOH; MeOH; MeOH:H_2_O:acetone (7:2:1), MeOH:H_2_O:acetone (7:1:2), and 70% aqueous acetone as eluents. The 50%MeOH fraction resulted in tellimagrandin I (**5**) (924 mg), which was purified over MCI GEL CHP-20P (30 × 1.1 cm i.d.) with aqueous MeOH and Mega Bond Elut C18 cartridge column with aqueous MeOH to yield pedunculagin (**4**) (148 mg) and 1,2,6-tri-*O*-galloyl-*β*-d-glucose (**7**) (143 mg). 1,2,3,6-Tetra-*O*-galloyl-*β*-d-glucose (**8**) (104 mg) and tellimagrandin II (**6**) (30 mg) was obtained from the 60% and 100% MeOH fractions, respectively. The MeOH:H_2_O:acetone (7:1:2) fraction yielded eucalbanin C (**9**) (682 mg) and a novel ellagitannin: eucarpanin D_2_ (**1**) (175 mg). The 70% aqueous acetone fraction resulted in eucamalin A (**2**) (37 mg). A portion of the *n*-BuOH extract (6.3 g) was chromatographed over Diaion HP-20 (70 × 1.5 cm i.d.) using H_2_O; 10%, 30%, and 50% aqueous MeOH; MeOH; and 70% aqueous acetone as eluents. The 30% MeOH fraction (1.5 g) was subjected to column chromatography over Toyopearl HW-40 (coarse grade) (40 × 2.2 cm i.d.) using 30%, 40%, 50%, 60%, and 70% aqueous MeOH; MeOH; MeOH:H_2_O:acetone (7:2:1), MeOH:H_2_O:acetone (7:1:2), and 70% aqueous acetone as eluents to give 1,6-di-*O*-galloyl-*β*-d-glucose (**10**) (20 mg), strictinin (**11**) (28 mg), compounds **4** (44 mg) and **5** (53 mg), and oenothein B (**12**) (360 mg). The water-soluble extract (120 g) was chromatographed over Diaion HP-20 (60 × 5 cm i.d.) using H_2_O; 10%, 30%, and 50% aqueous MeOH; MeOH; and 70% aqueous acetone as eluents. The 50% MeOH fraction (19 g) was further chromatographed over Toyopearl HW-40 (coarse grade) (40 × 2.2 cm i.d.) using 30%, 40%, 50%, 60%, and 70% aqueous MeOH; MeOH; MeOH:H_2_O:acetone (7:2:1); MeOH:H_2_O:acetone (7:1:2); and 70% aqueous acetone as eluents. The 60%MeOH fraction purified over MCI GEL CHP-20P (30 × 1.1 cm i.d.) with aqueous MeOH yielded Eugeniflorin D_1_ (**13**) (39 mg). For further ellagitannin studies, the leaves of *E. camaldulensis* were extracted again. The frozen leaves of *E. camaldulensis* (1.6 kg) were homogenized in 70% aqueous acetone (4.0 L × 3), filtered, and evaporated in vacuo. Extracts were obtained sequentially with Et_2_O (2.0 L × 3), EtOAc (2.0 L × 3), and water-saturated *n*-BuOH (2.0 L × 3) to produce Et_2_O (2.3 g), EtOAc (12.3 g), *n*-BuOH (31.8 g), and a water-soluble fraction (92.4 g). The EtOAc extract (10.0 g) was subjected to column chromatography over Toyopearl HW-40 (coarse grade) (40 × 2.2 cm i.d.) using 30%, 40%, 50%, 60%, and 70% aqueous MeOH; MeOH; MeOH:H_2_O:acetone (7:2:1); MeOH:H_2_O:acetone (7:1:2); and 70% aqueous acetone as eluents. The 50% MeOH fraction produced compound **5** (758 mg); the MeOH:H_2_O:acetone (7:1:2) fraction resulted in compounds **1** (340 mg) and **9** (677 mg); and the 70% aqueous acetone fraction produced compound **2** (20 mg). A portion of the *n*-BuOH extract (31.8 g) was separated by column chromatography over Diaion HP-20 (20 × 8 cm i.d.) using H_2_O with increasing amounts of MeOH (H_2_O, 10%, 20%, 40% and 100% MeOH) in a stepwise mode followed by 70% aqueous acetone. The 50% MeOH fraction (1.0 g) was chromatographed over Toyopearl HW-40 (coarse grade) (30 × 2.2 cm i.d.) with 30%, 40%, 50%, 60%, and 70% aqueous MeOH; MeOH:H_2_O:acetone (7:2:1); MeOH:H_2_O:acetone (7:1:2); and 70% aqueous acetone. The 50% aqueous MeOH fraction produced compound **12** (1.0 g) and was purified by MCI GEL CHP-20P (30 × 1.1 cm i.d.) and preparative reversed-phase HPLC to yield eurobustin C (**3**) (14 mg) and eugeniflorin D_2_ (**14**) (35 mg).

The physicochemical data obtained for compounds **1**–**3**, **9**, **13**, and **14** are provided below.

*Eucarpanin D_2_* (**1**): pale brown amorphous powder; [*a*${]}_{D}^{23}$ + 24.5° (*c* 0.002, MeOH); UV (MeOH) λ_max_ (log ε) 218 (5.17), 275 (4.83) nm; CD (MeOH) [θ] (nm) + 1.6 × 10^5^ (225), +1.7 × 10^5^ (237), −8.7 × 10^5^ (262), +7.9 × 10^5^ (285), −1.4 × 10^5^ (322); ^1^H-NMR [600 MHz, acetone-*d*_6_-D_2_O (9:1)] δ 7.11, 7.02, 7.02, 7.01, 6.10, 6.99 (2H, s, galloyl-H), 6.65, 6.64, 6.49, 6.48 (1H, s, HHDP-H), 6.87, 6.84, 6.60, 6.59, 6.53, 6.51 (1H, s, tergalloyl-H), 6.18 (1H, d, *J* = 8.4 Hz, Glc (I) H-1), 5.87 (1H, t, *J* = 9.6 Hz, Glc (II) H-3*α*), 5.85 (1H, t, *J* = 10.6 Hz, Glc (I) H-3), 5.67 (1H, t, *J* = 9.6 Hz, Glc (II) H-3*β*), 5.63 (1H, dd, *J* = 1.2, 8.4 Hz, Glc (I) H-2), 5.60 (1H, d, *J* = 5.4 Hz, Glc (II) H-1*α*), 5.33–5.23 (1H, m, Glc (I) H-4, H-6, Glc (II) H-2*β*, 6*αβ*), 5.16–5.11 (1H, m, Glc (II) H-2*α*, 4*αβ*), 5.00 (1H, d, *J* = 7.8 Hz, Glc (II) H-1*β*), 4.66 (1H, m, Glc (II) H-5*α*), 4.57 (1H, m, Glc (I) H-5), 4.26 (1H, m, Glc (II) H-5*β*), 3.93 (1H, brd, *J* = 13.2 Hz, Glc (I) H-6), 3.89–3.87 (2H, d, *J* = 12.6 Hz, Glc (II) H-6*αβ*); ^13^C-NMR [151 MHz, acetone-*d*_6_-D_2_O] δ 96.3 (Glc (II) C-1*β*), 93.5 (Glc (I) C-1), 90.8 (Glc (II) C-1*α*), 74.8 (Glc (II) C-2*β*), 73.5 (Glc (II) C-2*α*, 3*β*), 73.1 (Glc (II) C-3*α*), 72.7 (Glc (II) C-5*α*), 71.9 (Glc (II) C-5*β*), 71.8 (Glc (I) C-2), 71.3 (Glc (I) C-3), 71.0 (Glc (I) C-4, Glc (II) 3C-4*β*), 71.0 (Glc (II) C-4*α*), 67.0 (Glc (I) C-5), 63.4 (Glc (I) H-6, Glc (II) C-6*αβ*), 168.5, 168.0, 168.0, 167.8, 167.6, 167.2, 166.6, 166.0, 165.1 (ester carbonyl-C); HR-ESI-MS: *m/z* 1721.1649 [M − H]^−^, 860.0840 [M − 2H]^2−^ (calcd for C_75_H_54_O_48_ − H, 1721.1712).

Absolute configuration of glucopyranose. A solution of compound **1** (5.0 mg) in 5% H_2_SO_4_ was heated in boiling water for 6 h. The reaction mixture was purified using Sep-Pack Plus tC18 cartridges with H_2_O and MeOH. The aqueous layer was neutralized with Amberlite IRA-400 (OH^−^ form) and the resin was removed by filtration. After the removal of the solvent in vacuo, the residue was heated with l-cysteine methyl ester hydrochloride (0.5 mg) in pyridine (0.5 mL) to 60 °C for 1 h. Subsequently, *O*-tolyl isothiocyanate (0.5 mg) in pyridine (0.1 mL) added to the reaction mixture and further heated to 60 °C for 1 h. After the reaction, the solution was analyzed via reversed-phase HPLC (system A) (*t*_R_: 9.8 min).

Isomerization of compound **1** to eucarpanin D_1_ (**15**): A solution of compound **1** (1.0 mg) in the 0.02 M phosphate buffer (pH 7.4) (1.0 mL) was left standing at room temperature for 6 h. The isomerized product was identical to compound **15** according to the results of reversed-phase HPLC (system B) (*t*_R_: 10.13 min, 10.39 min).

Enzymatic hydrolysis of compound **1**: A solution of compound **1** (1.0 mg) in H_2_O (1.0 mL) was incubated with tannase (*Aspergillus ficuum*) at 37 °C for 2 h. The product was identified as compound **9** using reversed-phase HPLC (system B) (*t*_R_: 7.02 min, 8.11 min).

Partial hydrolysis of compound **1**: A solution of compound **1** (20 mg) in water was heated to 98 °C for 5 h. The reaction mixture was purified using Sephadex LH-20 (30 × 1.0 cm i.d.) and MCI GEL CHP-20P (30 × 1.0 cm i.d.) and subjected to reversed-phase HPLC (system B) to reveal the presence of compounds **5** (1.6 mg), **6** (3.5 mg), and **17** (1.5 mg) in the hydrolysate.

Methylation of compound **1** followed by methanolysis: A mixture of compound **1** (20 mg), potassium carbonate (200 mg), unhydrate, and dimethyl sulfate (100 µL) in super dehydrate acetone (2.0 mL) was stirred and refluxed for 6 h. After removing the inorganic material via centrifugation, the supernatant was evaporated. The reaction mixture was directly methanolyzed in 1% sodium methoxide (0.5 mL) and superdehydrated MeOH (0.1 mL) and left standing overnight at room temperature. After adding acetic acid (two drops), the solution was evaporated under nitrogen gas. The solution was treated with excess diazomethane (0.8 mL) and Et_2_O (0.4 mL) for 3 h and then evaporated in vacuo. The residue was subjected to preparative TLC (TLC Silica gel 60 PF_254_, Merck KGaA) with toluene:acetone (4:1) to compounds **18** (6.0 mg), **19** (1.5 mg), and **20** (1.7 mg). All three materials were characterized by HR-ESI-MS, normal-phase HPLC (system D), and CD spectroscopy, which revealed that these compounds were identical to the authentic samples. Compound **19**: CD (MeOH) [θ] (nm) + 5.3 × 10^4^ (229), −4.0 × 10^4^ (252), +6.9 × 10^3^ (313); HR-ESI-MS: *m/z* 473.1419 [M + Na]^+^ (calcd for C_22_H_26_O_10_ + Na, 473.1418); normal-phase HPLC (system D): *t*_R_ 6.1 min. Compound **20**: CD (MeOH) [θ] (nm) + 4.6 × 10^4^ (221), −3.6 × 10^4^ (254), +7.5 × 10^3^ (317); HR-ESI-MS: *m/z* 683.1937 [M + Na]^+^ (calcd for C_32_H_36_O_15_ + Na, 683.1946); normal-phase HPLC (system D): *t*_R_ 6.5 min.

*Eucamalin A* (**2**): pale brown amorphous powder; [*a*${]}_{D}^{25}$ + 49.2° (*c* 0.002, MeOH); UV (MeOH) λ_max_ (log ε) 218 (5.33) nm, 272 (4.50) nm; CD (MeOH) [θ] (nm) + 2.9 × 10^5^ (222), +2.7 × 10^5^ (237), −1.7 × 10^5^ (262), +1.4 × 10^5^ (286), −2.5 × 10^4^ (323); ^1^H-NMR [400 MHz, acetone-*d*_6_-D_2_O (9:1)] δ 7.06–6.96 (2H, s, galloyl-H), 6.87–6.83 (1H, s, tergalloyl-H), 6.65–6.62 (1H, s, HHDP-H), 6.60–6.56 (1H, s, Tergalloyl-H), 6.52–6.47 (1H, s, tergalloyl and HHDP-H), 5.90–5.82 (m, Glc H-3*αβ*), 5.67 (t, *J* = 9.6 Hz, Glc H-3*β*), 5.63–5.54 (m, Glc H-1*α*), 5.56 (d, *J* = 3.6 Hz, Glc H-1*α*), 5.28–5.22 (m, Glc H-2*α*, 6*αβ*), 5.16–5.09 (m, Glc H-1*β*, 2*α*, 4), 5.00 (d, *J* = 7.2 Hz, Glc H-1*β*), 4.70–4.64 (m, Glc H-5*α*), 4.30–4.24 (m, Glc H-1*β*, H-5*β*), 3.91–3.79 (Glc H-6*αβ*); ^13^C-NMR [100 MHz, acetone-*d*_6_-D_2_O (9:1)]: δ 96.6, 96.4 (Glc C-1*β*), 91.1, 90.8 (Glc C-1*α*), 75.1 (Glc C-2*β*), 74.1 (Glc C-2*β*), 73.7 (Glc C-3, 4), 73.5 (Glc C-2*α*), 73.0 (Glc C-2*α*), 71.9, 71.7 (Glc C-5*β*), 71.2 (Glc C-3*αβ*), 71.0 (Glc C-4), 67.0, 66.91 (Glc C-5*α*), 63.9, 63.4 (Glc C-6*αβ*), 168.3, 168.1, 167.8, 167.8, 169.0, 166.9, 166.6, 166.3, 165.9 (ester carbonyl-C); HR-ESI-MS: *m/z* 2353.2400 [M − H]^−^, 1176.1117 [M − 2H]^2−^, 783.7383 [M − 3H]^3−^.

Absolute configuration of glucopyranose. A solution of compound **2** (2.0 mg) in 5% H_2_SO_4_ was heated in boiling water for 1 h. The reaction mixture was purified using Sep-Pack Plus tC18 cartridges with H_2_O and MeOH. The aqueous layer was neutralized with Amberlite IRA-400 (OH^−^ form) and the resin was removed by filtration. After removal of the solvent in vacuo, the residue was heated with l-cysteine methyl ester hydrochloride (0.5 mg) in pyridine (0.5 mL) to 60 °C for 1 h. Subsequently, *O*-tolyl isothiocyanate (0.5 mg) in pyridine (0.1 mL) added to the reaction mixture and further heated to 60 °C for 1 h. After the reaction, the solution was analyzed via reversed-phase HPLC (system A) (*t*_R_: 9.8 min).

Isomerization of compound **2** to eucarpanin T_1_ (**16**): A solution of compound **2** (4.0 mg) in the 0.02 M phosphate buffer (pH 7.4) (2.0 mL) was stored at room temperature for 9 h. The isomerized product was identical to compound **16** according to ^1^H-NMR.

Partial hydrolysis of compound **2**: A solution of compound **2** (10 mg) in water was heated to 98 °C for 2 h. The reaction mixture was purified by MCI GEL CHP-20P (20 × 1.0 cm i.d.) and subjected to reversed-phase HPLC (system B) to reveal the presence of compounds **5** (0.5 mg) and **17** (0.7 mg) in the hydrolysate.

Methanolysis of compound **2**: A mixture of compound **2** (5.0 mg), potassium carbonate (100 mg), unhydrate, and dimethyl sulfate (100 µL) in super dehydrated acetone (1.0 mL) was stirred and refluxed for 6 h. After removing the inorganic material via centrifugation, the supernatant was evaporated. The reaction mixture was directly methanolyzed in 1% sodium methoxide (0.5 mL) and superdehydrated MeOH (0.1 mL) and left standing overnight at room temperature. After adding acetic acid (two drops), the solution was evaporated under nitrogen gas. The solution was treated with an excess of diazomethane (0.4 mL) and Et_2_O (0.2 mL) for 3 h and then evaporated in vacuo. The residue was subjected to preparative TLC (TLC Silica gel 60 PF_254_, Merck KGaA) with toluene: acetone (4:1) to yield compounds **18** (1.6 mg), **19** (1.3 mg), and **20** (0.9 mg). All three compounds were characterized by HR-ESI-MS and CD spectroscopy, which revealed that they were identical to the authentic samples. Compound **18**: HR-ESI-MS: *m/z* 227.0968 [M + H]^+^ (calcd for C_11_H_15_O_5_ + H: 227.0919). Compound **19**: CD (MeOH) [θ] (nm) + 5.3 × 10^4^ (228), −4.1 × 10^4^ (252), +7.1 × 10^3^ (313); HR-ESI-MS: *m/z* 450.1517 [M]^+^ (calcd for C_22_H_26_O_10_, 450.1526); normal-phase HPLC (system D): *t*_R_ 6.1 min. Compound **20**: CD (MeOH) [θ] (nm) + 5.0 × 10^4^ (220), −4.0 × 10^4^ (254), +8.6 × 10^3^ (314); HR-ESI-MS: *m/z* 661.2113 [M + H]^+^ (calcd for C_32_H_35_O_15_ + H, 661.2132); normal-phase HPLC (system D): *t*_R_ 6.4 min.

*Eurobustin C* (**3**): pale brown amorphous powder; [*α*${]}_{D}^{23}$ + 13° (*c* 1, MeOH); UV (MeOH) λ_max_ (log ε) 219 (4.94), 685 (4.69) nm; CD (MeOH) [θ] (nm) − 2.4×10^4^ (206), +1.9 × 10^5^ (218), +8.4 × 10^4^ (228), +2.0 × 10^5^ (240), −1.3 × 10^5^ (263), +5.7 × 10^4^ (284), −1.7 × 10^4^ (308); ^1^H-NMR [400 MHz, acetone-*d*_6_-D_2_O (9:1)] δ 7.15, 7.08 (2H, s, galloyl-H), 7.20, 6.85, 6.56 (1H, s, valoneoyl-H), 6.39, 6.26 (1H, s, eurobustinoyl-H), 5.94 (1H, d, *J* = 3.6 Hz, Glc (I) H-1*α*), 5.93 (1H, t, *J* = 4.0 Hz, Glc (I) H-2), 5.92 (1H, t, *J* = 9.6 Hz, Glc (I) H-3*α*), 5.42 (1H, t, *J* = 8.8 Hz, Glc (II) H-3*β*), 5.21 (1H, dd, *J* = 6,13 Hz, Glc (I) H-6*α*), 5.08 (1H, dd, *J* = 6.4, 13.2 Hz, Glc (II) H-6*β*), 4.99 (1H, t, *J* = 10.0 Hz, Glc (I) H-4*α*), 4.90 (1H d, *J* = 8.8 Hz, Glc (II) H-1*β*), 4.82 (1H, t, *J* = 8.8 Hz, Glc (II) H-2*β*, 4*β*), 4.80 (1H, dd, *J* = 6.4, 10.0 Hz, Glc (I) H-5*α*), 4.45 (1H, dd, *J* = 2.0, 8.0 Hz, methin-H), 3.65 (2H, d, *J* = 13.2 Hz, Glc (II) H-6*β*), 3.57 (2H, d, *J* = 12.8 Hz, Glc (I) H-6*α*), 2.14 (1H, dd, *J* = 1.6, 18.8 Hz, methylene-H), 1.89 (1H, dd, *J* = 8.0, 19.2 Hz, methylene-H); ^13^C-NMR [100 MHz, acetone-*d*_6_-D_2_O] δ 96.2 (Glc (II) C-1), 91.9 (Glc (I) C-1), 74.7 (Glc (I) C-2), 73.9 (Glc (II) C-2), 73.3 (Glc (II) C-3), 72.1 (Glc (II) C-4), 70.6 (Glc (I) C-4), 70.4 (Glc (II) C-5), 69.8 (Glc (I) C-3),68.2 (Glc (I) C-5), 63.2, 62.9 (Glc (I) (II) C-6), 170.5, 168.2, 167.9, 167.6, 166.9, 166.2, 165.0 (ester carbonyl-C), 197.2 (ketone-C); HR-ESI-MS: *m/z* 1539.1423 [M − H]^−^, 769.0657 [M − 2H]^2−^ (calcd for C_67_H_48_O_43_ − H, 1539.1491).

Partial hydrolysis of compound **3**: A solution of compound **3** (0.7 mg) in hot water containing 1% TFA (0.7 mL) was heated in a water bath for 5 h. The reaction mixture was subjected to reversed-phase HPLC (System C) to reveal the presence of compounds **21**–**25** in the hydrolysate.

Methylation of compound **3** followed by methanolysis: diazomethane-EtOH (1 mL) was added to an EtOH solution (0.5 mL) of compound **3** (10 mg), and the obtained mixture was stored for 3 h at room temperature. After removing the solvent using an evaporator, a MeOH solution (1.0 mL) of the residue was added to 1% sodium methoxide (20 drops) and left standing overnight at room temperature. After the addition of AcOH, the solution was evaporated, and the residue was partitioned between EtOAc and H_2_O. An EtOAc layer was added, the residue was further treated with diazomethane (10 drops)–Et_2_O for 2 h, and the solvent was evaporated. The residue was subjected to preparative thin layer chromatography (TLC) (TLC silica gel 60 PF_254_, Merck KGaA) with toluene:acetone (4:1) to yield compounds **18**, **19**, **26**, and **27**. All four materials were characterized via CD spectroscopy, which revealed that these compounds were identical to the authentic samples. Compound **26**: CD (MeOH) [θ] (nm) − 4.4 × 10^4^ (202), +1.5 × 10^5^ (220), −7.3 × 10^4^ (251), +2.0 × 10^3^ (278), −2.0 × 10^3^ (294), +1.7 × 10^4^ (315). Compound **27**: CD (MeOH) [θ] (nm) − 5.7 × 10^4^ (204), +1.1 × 10^5^ (228), −8.2 × 10^4^ (254), +1.0 × 10^4^ (278), −1.1 × 10^4^ (297), +5.1 × 10^3^ (316).

*Eucalbanin C* (**9**): pale brown amorphous powder; ^1^H-NMR [600 MHz, acetone-*d*_6_-D_2_O (9:1)] δ 7.06, 7.05, 7.05, 7.04, 7.02, 7.02, 7.01, 6.99, 6.99, 6.96 (2H in total each, s, galloyl-H), 6.65, 6.64, 6.64, 6.63, 6.49, 6.48 (1H, s, HHDP-H), 6.87, 6.86, 6.84, 6.83, 6.59, 6.58, 6.58, 6.52, 6.50, 6.50 (1H, s, tergalloyl-H), 5.86 (2H, t, Glc (I) H-3*α*, Glc (I) H-3β), 5.66 (1H, t, Glc (I) H-3*β*), 5.60 (2H, m, Glc (II) H-1α, H-3α), 5.54 (1H, d, *J* = 3.6 Hz, Glc (I) H-1α), 5.28–5.20 (1H, m, Glc (I) H-2*β*, Glc (II) H-2*β*, Glc (I) (II) H-6*αβ*), 5.17–5.07 (1H, m, Glc (I) H-2*α*, Glc (II) H-2*α*, Glc (I) (II) H-4*αβ*), 4.37 (1H, m, *J* = 7.8 Hz, Glc (II) H-1*β*), 4.98 (1H, d, *J* = 7.8 Hz, Glc (I) H-1*β*), 4.66 (2H, m, Glc (I) (II) H-5*α*), 4.26 (2H, m, Glc (I) (II) H-5*β*), 3.83 (2H, m, Glc (I) (II) H-6’*αβ*); ^13^C-NMR [151 MHz, acetone-*d*_6_-D_2_O] δ 96.5 (Glc (II) C-1*β*), 96.3 (Glc (I) C-1*β*), 91.1 (Glc (I) C-1*α*), 90.7 (Glc (II) C-1*α*), 74.8 (Glc (I) (II) C-2*β*), 74.0 (Glc (I) (II) C-2*α*), 73.7, 73.6 (Glc C-3*β*), 71.9, 71.7 (Glc (I) (II) C-5*β*), 71.4 (Glc (I) (II) C-3*α*), 73.0, 71.2, 71.1 (Glc (I) (II) C-4*β*), 71.0 (Glc (I) (II) C-4*α*), 66.9, 66.8 (Glc (I) (II) C-5*α*), 63.9, 63.5 (Glc (I) (II) C-6*αβ*); HR-ESI-MS: *m/z* 1569.1569 [M − H]^−^, 785.0816 [M − 2H]^2−^ (calcd for C_68_H_50_O_44_ − H, 1569.1602).

*Eugeniflorin D_1_* (**13**): pale brown amorphous powder; ^1^H-NMR [600 MHz, acetone-*d*_6_-D_2_O (9:1)] δ 6.94, 6.92, 6.91 (2H in total each, s, galloyl-H), 6.97, 6.82, 6.65, 6.61, 6.52, 6.33 (1H, s, valoneoyl-H), 6.40 (1H, d, *J* = 8.4 Hz, Glc (I) H-1), 5.77 (1H, t, *J* = 9.6 Hz, Glc (I) H-3), 5.40 (1H, dd, *J* = 7.2 Hz, Glc (II) H-6), 5.36 (1H, t, *J* = 9.0 Hz, Glc (II) H-3), 5.22–5.15 (3H, m, Glc (I) H-2, Glc (II) H-4, 6), 5.03 (1H, t, *J* = 9.0 Hz, Glc (II) H-2), 4.61–4.58 (1H, m, Glc (I) H-5), 4.42 (1H, d, *J* = 7.8 Hz, Glc (II) H-1), 4.38 (1H, dd, *J* = 6.0, 10.2 Hz, Glc (II) H-5), 4.00 (1H, dd, *J* = 2.4, 13.2 Hz, Glc (I) H-6), 3.91 (1H, d, *J* = 13.2 Hz, Glc (II) H-6’), 3.88–3.84 (1H, m, Glc (I) H-6’); ^13^C-NMR [151 MHz, acetone-*d*_6_-D_2_O] 95.8 (Glc (II) C-1), 93.0 (Glc (I) C-1), 75.6 (Glc (I) C-2), 74.5 (Glc (II) C-2), 73.9 (Glc (II) C-4), 72.1 (Glc (II) C-3), 72.0 (Glc (I) C-5), 71.8 (Glc (II) C-5), 71.7 (Glc (I) C-3), 71.3 (Glc (I) C-4), 66.0 (Glc (II) C-6), 63.1 (Glc (I) C-6), 170.4, 168.9, 168.8, 168.1, 166.9, 166.9, 165.9, 164.8 (ester carbonyl-C); HR-ESI-MS: *m/z* 1719.1532 [M − H]^−^, 859.0759 [M − 2H]^2−^ (calcd for C_75_H_52_O_28_ − H, 1719.1555).

*Eugeniflorin D_2_* (**14**): pale brown amorphous powder; ^1^H-NMR [400 MHz, acetone-*d*_6_-D_2_O (9:1)] δ 7.30, 7.16 (2H in total each, s, galloyl-H), 7.17, 6.77 (1H, s, dehydrovaloneoyl-H), 7.10 (1H, d, *J* = 2.0 Hz, dehydrovaloneoyl-H), 6.57, 6.36, 5.87 (1H, s, valoneoyl-H), 5.98 (1H, d, *J* = 3.2 Hz, Glc (I) H-1*α*), 5.90 (1H, t, *J* = 10.0 Hz, Glc (I) H-3*α*), 5.69 (1H, dd, *J* = 3.2, 10.0 Hz, Glc (I) H-2*α*), 5.55 (1H, t, *J* = 10.0 Hz, Glc (II) H-3*β*), 5.24 (1H, t, *J* = 8.0 Hz, Glc (II) H-2*β*), 5.19 (1H, d, *J* = 8.0 Hz, Glc (II) H-1*β*), 5.09 (1H, dd, *J* = 6.4, 13.6 Hz, Glc (II) H-6*β*), 4.91 (1H, t, *J* = 10.0 Hz, Glc (II) H-4*αβ*), 4.70 (1H, dd, *J* = 5.6, 12.8 Hz, Glc (I) H-6*α*), 4.49 (1H, dd, *J* = 5.6, 10.0 Hz, Glc (I) H-5*α*), 4.05 (1H, dd, *J* = 6.4, 10.0, Glc (II) H-5*β*), 3.74–3.70 (2H, m, Glc (I) H-6*α*, Glc (II) H-6*β*); ^13^C-NMR [100 MHz, acetone-*d*_6_-D_2_O] δ 96.2 (Glc (II) C-1*β*), 91.7 (Glc (I) C-1*α*), 75.4 (Glc (I) C-2*α*), 74.9 (Glc (II) C-2*β*), 72.5 (Glc (I) C-4*α*), 72.4 (Glc (II) C-3*β*), 71.3 (Glc (II) C-5*β*), 71.0 (Glc (I) C-4*α*), 70.3 (Glc (I) C-3*α*), 68.5 (Glc (I) C-5*α*), 64.4 (Glc (I) C-6*α*), 63.5 (Glc (II) C-6*β*), 168.4, 168.3, 167.4, 166.2, 164.5, 163.9 (ester carbonyl-C), 194.1 (ketone-C); HR-ESI-MS: *m/z* 1583.1389 [M − H]^−^, 791.0640 [M − 2H]^2−^ (calcd for C_68_H_48_O_45_ − H, 1583.148).

### 3.4. Bioassay for Detoxification Studies

The Al detoxification properties of the fractionated extracts and isolated ellagitannins were evaluated by a bioassay using the Al-sensitive model plant *Arabidopsis thaliana*, as described previously [7]. Briefly, pretreated *A. thaliana* seeds were germinated on culture equipment comprising glass slides and nylon mesh inside a growth chamber. Seedlings were grown hydroponically in a 2% strength-modified Molecular Genetics Research Laboratory (MGRL) medium (without phosphate ions and with 200 μM CaCl_2_ instead of calcium salts). After 4 d of culture, the seedlings were treated with a modified MGRL medium (pH 5.0) containing 0 or 12 μM AlCl_3_ and 0, 8, or 16 μM ellagitannin for 24 h [30]. To evaluate the detoxification properties of the extracts, 3 mg of each dried extract was added to 500 mL of the medium instead of the ellagitannin. Images of the root were taken with a scanner before and after treatment and expressed relative to controls (0 µM Al, 0 µM ellagitannin). The elongation of each primary root was measured using the WinRHIZO Pro image analysis software (https://regent.qc.ca/assets/winrhizo_software.html, Régent Instruments, Quebec, QC, Canada).

## 4. Conclusions

In summary, we isolated two undescribed ellagitannin oligomers, i.e., eucarpanin D_2_ (**1**, dimer) and eucamalin A (**2**, trimer), along with 14 known compounds from the leaves of *E. camaldulensis*, including gallotannins (**7**, **8** and **10**), monomeric ellagitannins (**4**–**6** and **11**), and dimeric ellagitannins (**3**, **9**, **12** and **14**). Compounds **1**–**3** were identified via HR-ESI-MS, NMR, chemical reactions, and physicochemical data. The detailed chemical structure of **3** was elucidated for the first time. Furthermore, the ellagitannins obtained from *E. camaldulensis*, especially compound **2** and eugeniflorin D_1_ (**13**), demonstrated high abilities to alleviate Al toxicity. These results suggest that ellagitannins other than oenothein B (**12**) possess good Al detoxification properties. Therefore, further studies should focus on ellagitannins that are present not only in the leaves, but also in the roots of *E. camaldulensis*. Although further studies are required to achieve a better understanding of the Al detoxification mechanism of ellagitannins, these results suggest that these tannins promote the growth of *E. camaldulensis* in acidic soils.

## Data Availability

Data will be made available on request.

## Supplementary Materials

The following materials are available online: https://www.mdpi.com/article/10.3390/molecules30102216/s1, Figure S1: Separation procedure of leaves of E. camaludulensis. First time (A), second time (B). Figure S2: 1D- and 2D-NMR spectra of eucarpanin D2 (1). 1H-NMR (A), 13C-NMR (B), COSY (C), HSQC (D), and HMBC (E) spectrum. Figure S3: 1D-NMR spectra of eucamalin A (2). 1H-NMR (A), 13C-NMR (B), COSY (C), HSQC (D), and HMBC (E) spectrum. Figure S4: 1D-NMR spectra of eurobustin C (3). 1H-NMR (A), 13C-NMR (B), COSY (C), HSQC (D), and HMBC (E) spectrum.

## Abbreviations

The following abbreviations are used in this manuscript:

HPLC	High-performance liquid chromatography
HR–ESI–MS	High-resolution electrospray ionization mass spectrometry
NMR	Nuclear magnetic resonance
HHDP	Hexahydroxydiphenoyl
COSY	Correlation spectroscopy
HMBC	Heteronuclear multiple bond correlation
CD	Circular dichroism
UV	Ultraviolet
HSQC	Heteronuclear single quantum correlations
TLC	Thin layer chromatography
