# Supplementary material for: Ellagitannin Oligomers from Eucalyptus camaldulensis Leaves and Their Role in the Detoxification of Aluminum
Source: Molecules. 2025 May 19;30(10):2216. doi: 10.3390/molecules30102216 (PMC12114028; doi:10.3390/molecules30102216)
Supplement: Supplementary file 1 [file molecules-30-02216-s001.zip › molecules-3616903-supplementary.pdf]

Ellagitannin Oligomers from *Eucalyptus camaldulensis* Leaves and Their Role in the Detoxification of Aluminum

Haruna Uemori 1,2, Ayano Inoue 3, Shoichi Suzuki 3, Yuji Iwaoka 1, Tsutomu Hatano 4, Morio Yoshimura 5, Yoshiaki Amakura 5, Toshiyuki Murakami 2, Ko Tahara 6 and Hideyuki Ito 1,7,*

1 Doctorate Course of Health and Welfare Science, Graduate School of Okayama Prefectural University, 111 Kuboki, Soja 719-1197, Okayama, Japan;
h-uemori@maruzenpcy.co.jp (H.U.); iwaoka@fhw.oka-pu.ac.jp (Y.I.);
2 Research Center, Maruzen Pharmaceuticals, Co., Ltd., 1089-8 Sagata, Shinnichi-Cho, Fukuyama 729-3102, Hiroshima, Japan; to-murakami@maruzenpcy.co.jp
3 Faculty of Health and Welfare Science, Okayama Prefectural University, 111 Kuboki, Soja 719-1197, Okayama, Japan; ayano-.-10251@outlook.jp (A.I.);
ssho5532@gmail.com (S.S.)
4 Division of Pharmaceutical Sciences, Graduate School of Medicine, Dentistry, Pharmaceutical Sciences, Okayama University, 1-1-1 Tsushima-Naka, Kita-ku 700-8530, Okayama, Japan; hatano-t@cc.okayama-u.ac.jp
5 Pharmaceutical Sciences, Matsuyama University, 4-2 Bunkyo-cho, Matsuyama 790-8578, Ehime, Japan;
myoshimu@g.matsuyama-u.ac.jp (M.Y.);
amakura@g.matsuyama-u.ac.jp (Y.A.)
6 Forestry and Forest Products Research Institute, 1 Matunosato, Tsukuba 305-8687, Ibaraki, Japan;
taharako@affrc.go.jp
7 National Institutes of Biomedical Innovation, Health and Nutrition, Kento Innovation Park, NK Building, 3-17 Senrioka, Shinmachi 566-0002, Osaka, Japan
* Correspondence: ito-hideyuki@nibn.go.jp

Supporting Information

Figure S1: Separation procedure of leaves of *E. camaludulensis*. First time (A), second time (B).

Figure S2: 1D and 2D-NMR spectra of eucarpanin D_2 (**1**). ^{1}H-NMR (A), ^{13}C-NMR (B), COSY (C), HSQC (D), and HMBC (E) spectrum.

Figure S3: 1D-NMR spectra of eucamalin A (**2**). ^{1}H-NMR (A), ^{13}C-NMR (B), COSY (C), HSQC (D), and HMBC (E) spectrum.

Figure S4; 1D-NMR spectra of eurobustin C (**3**). ^{1}H-NMR (A), ^{13}C-NMR (B), COSY (C), HSQC (D), and HMBC (E) spectrum.

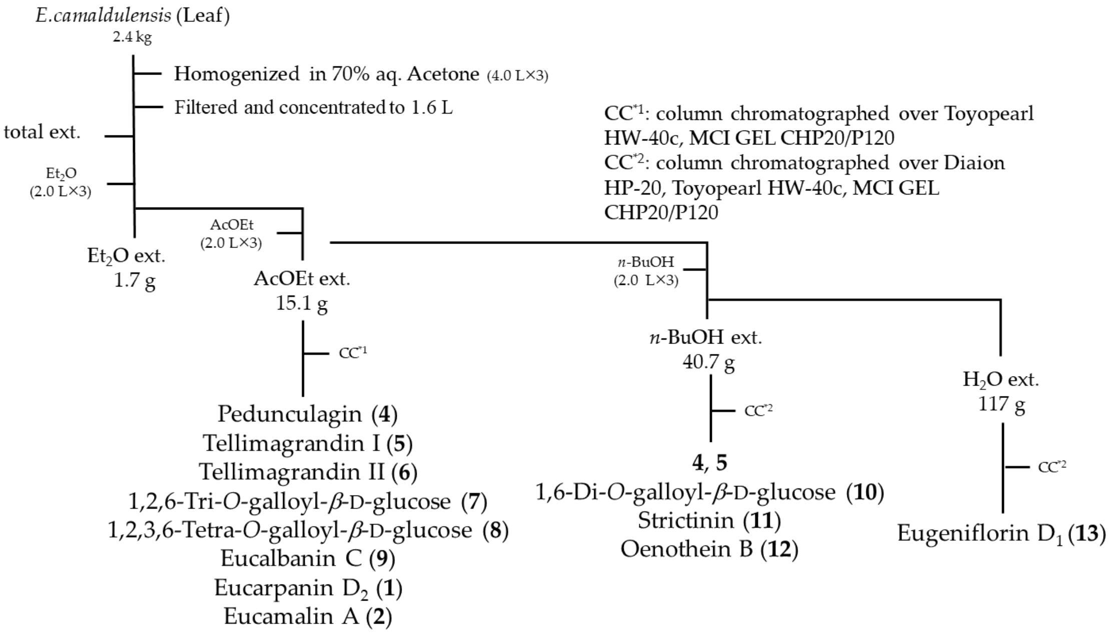

Figure S1A

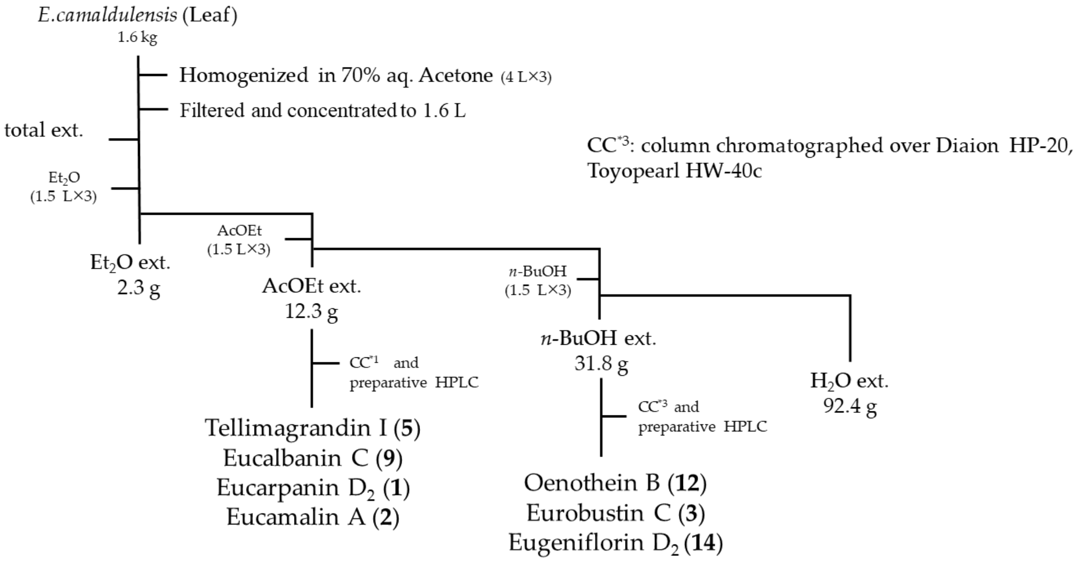

Figure S1B

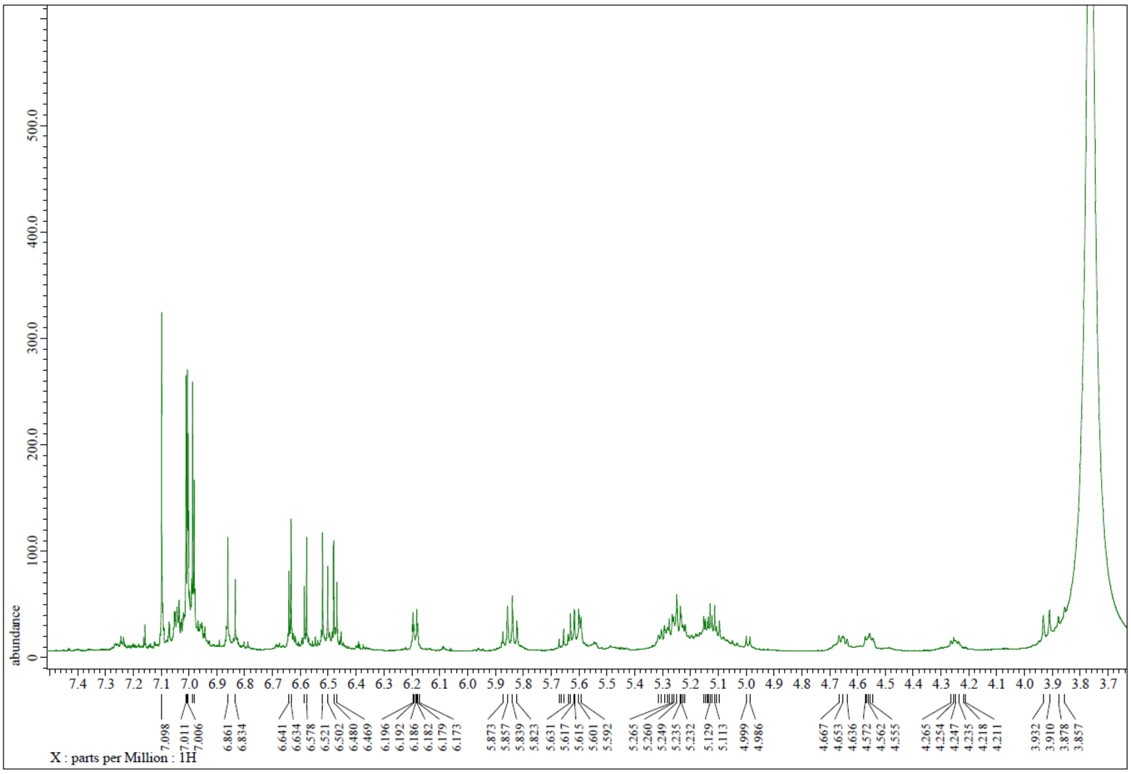

Figure S2A

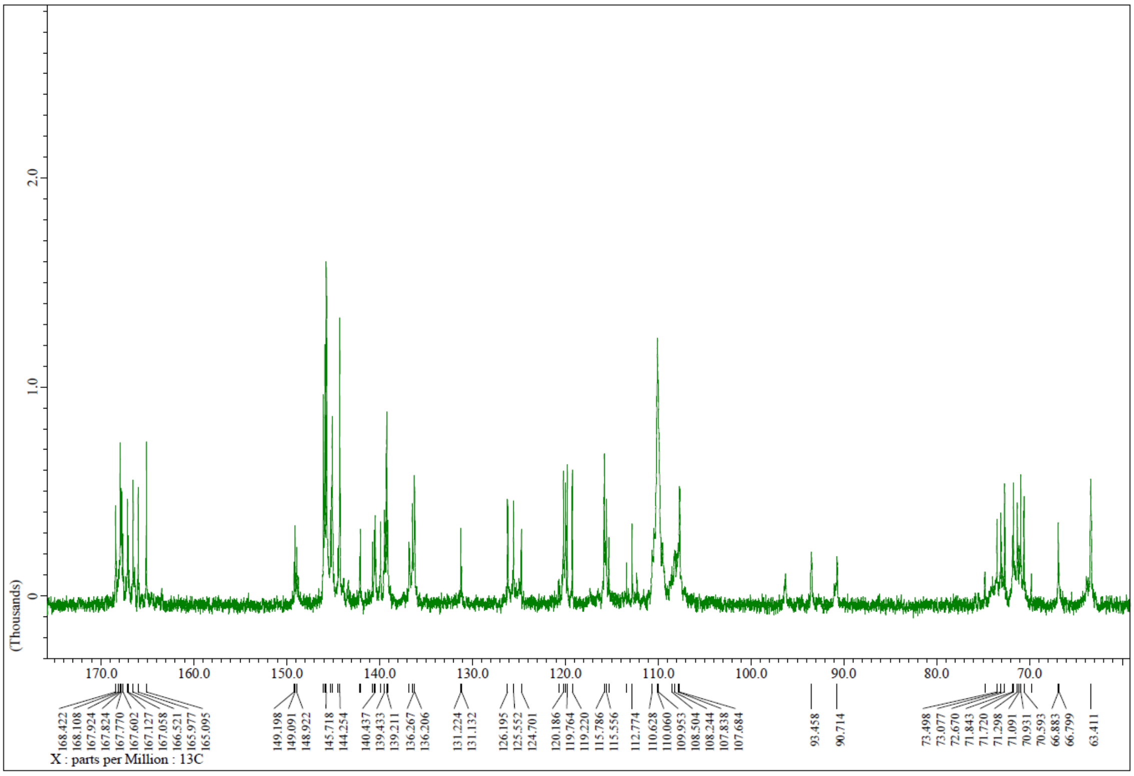

Figure S2B

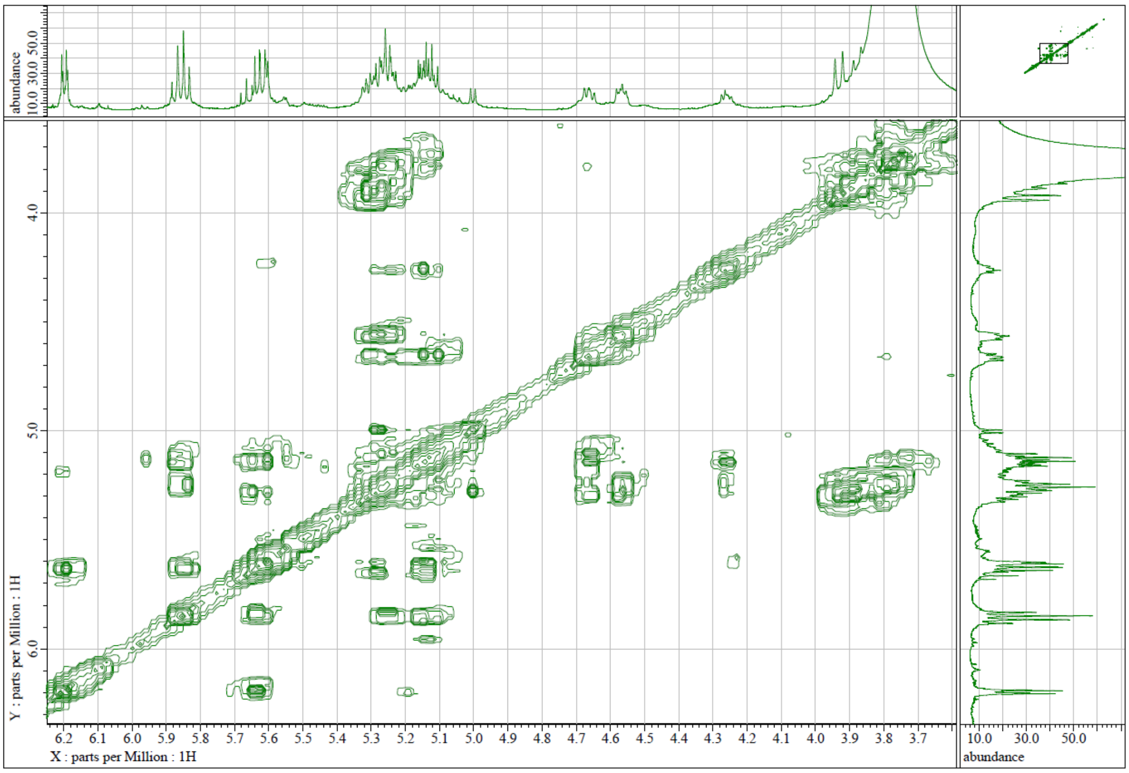

Figure S2C

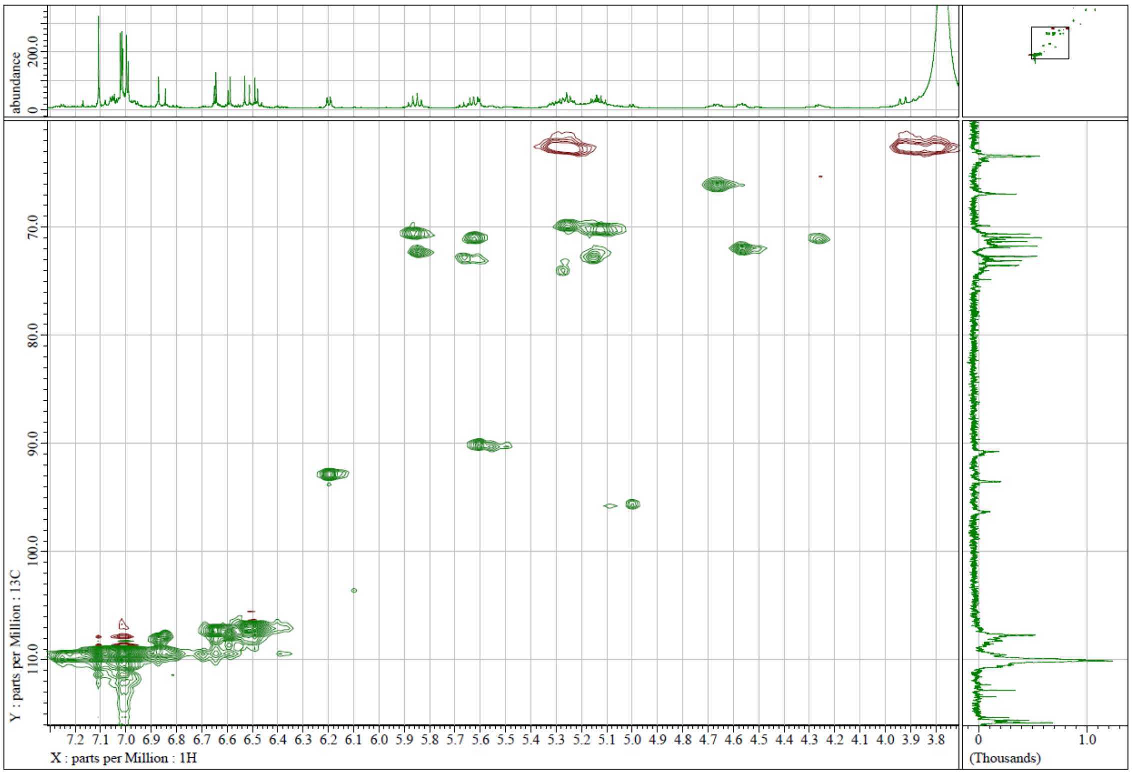

Figure S2D

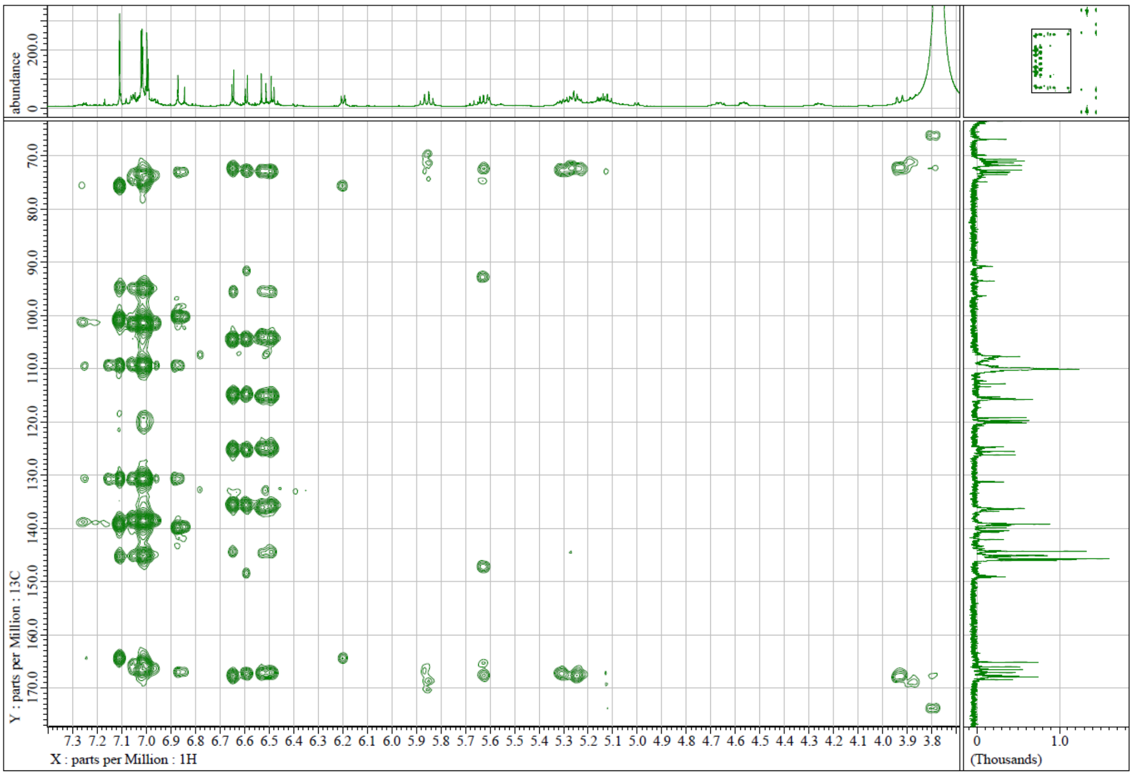
abundance
Y : parts per Million : 13C
X : parts per Million : 1H
(Thousands)

Figure S2E

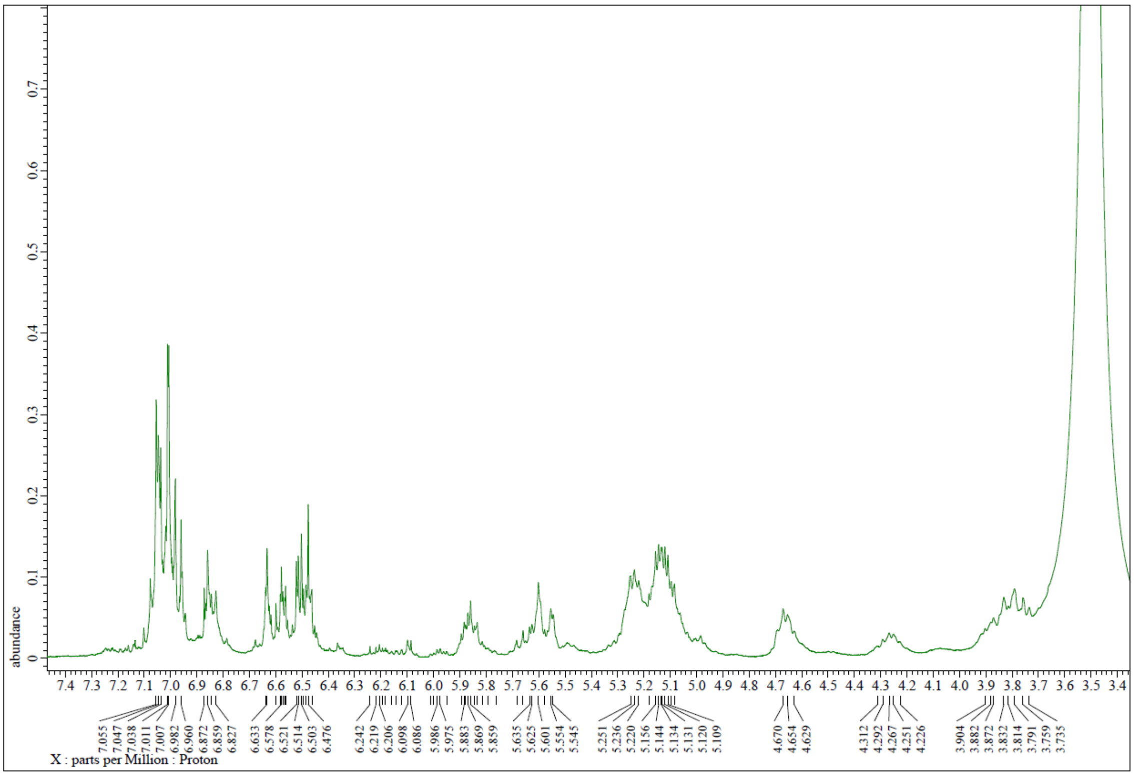

Figure S3A

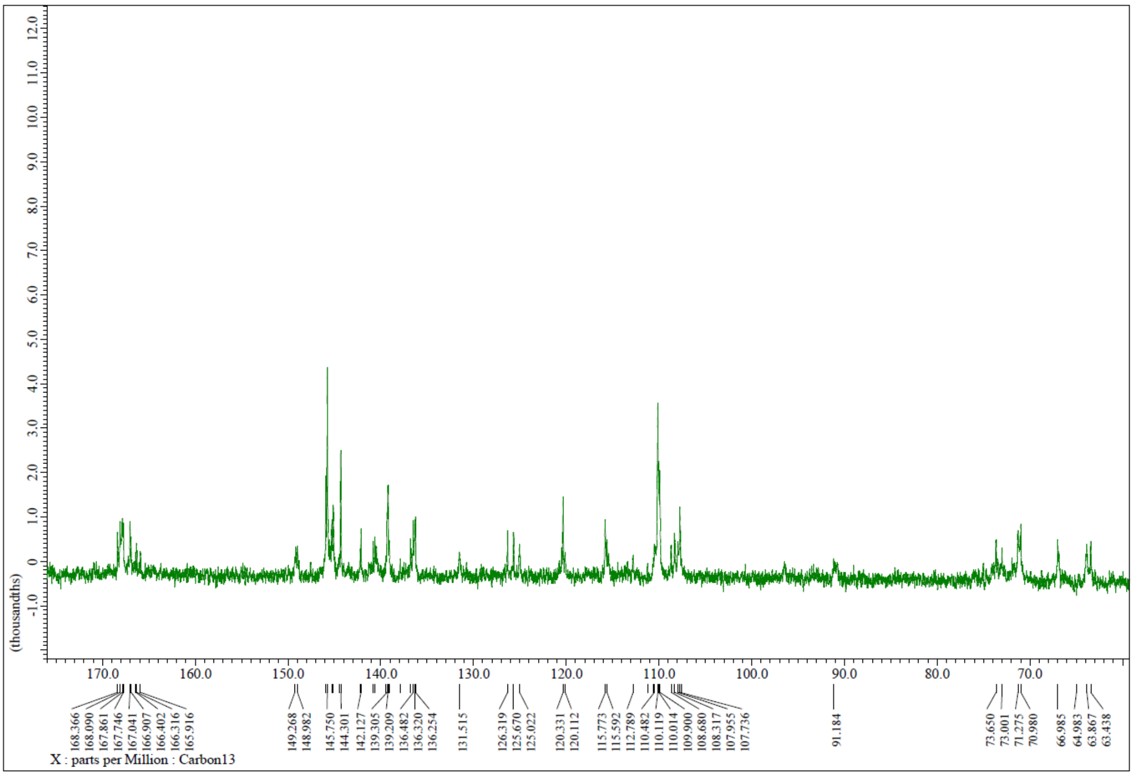

Figure S3B

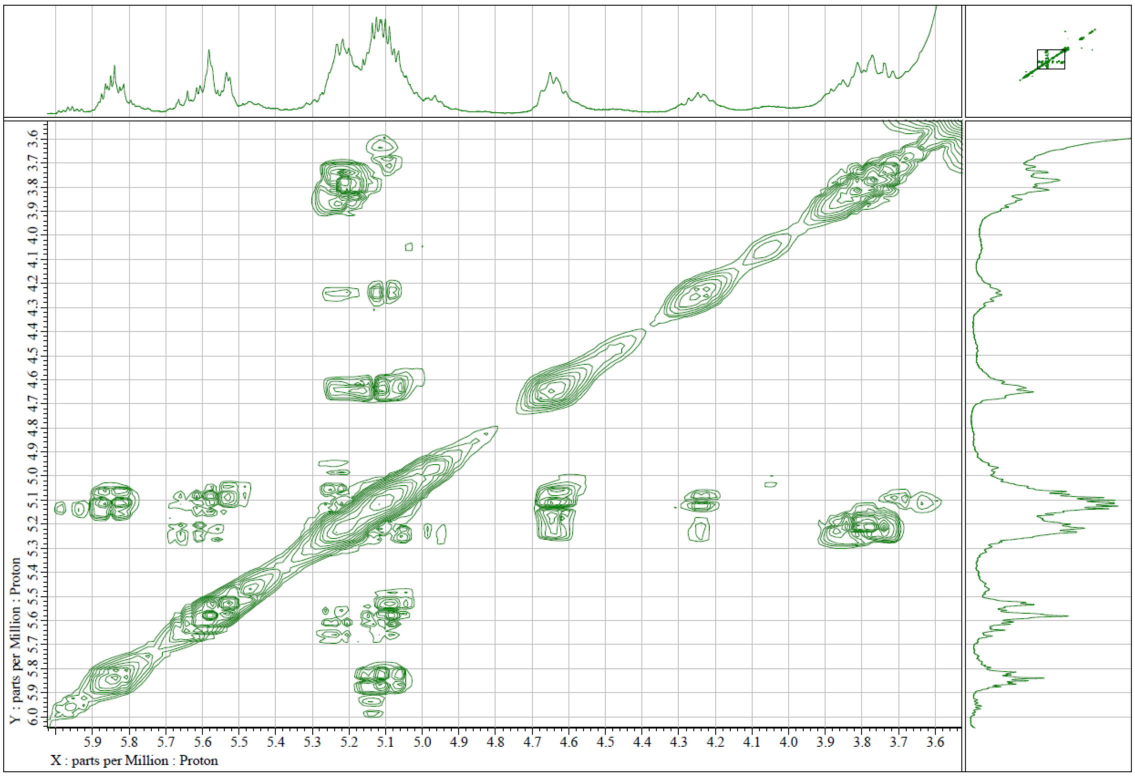

Figure S3C

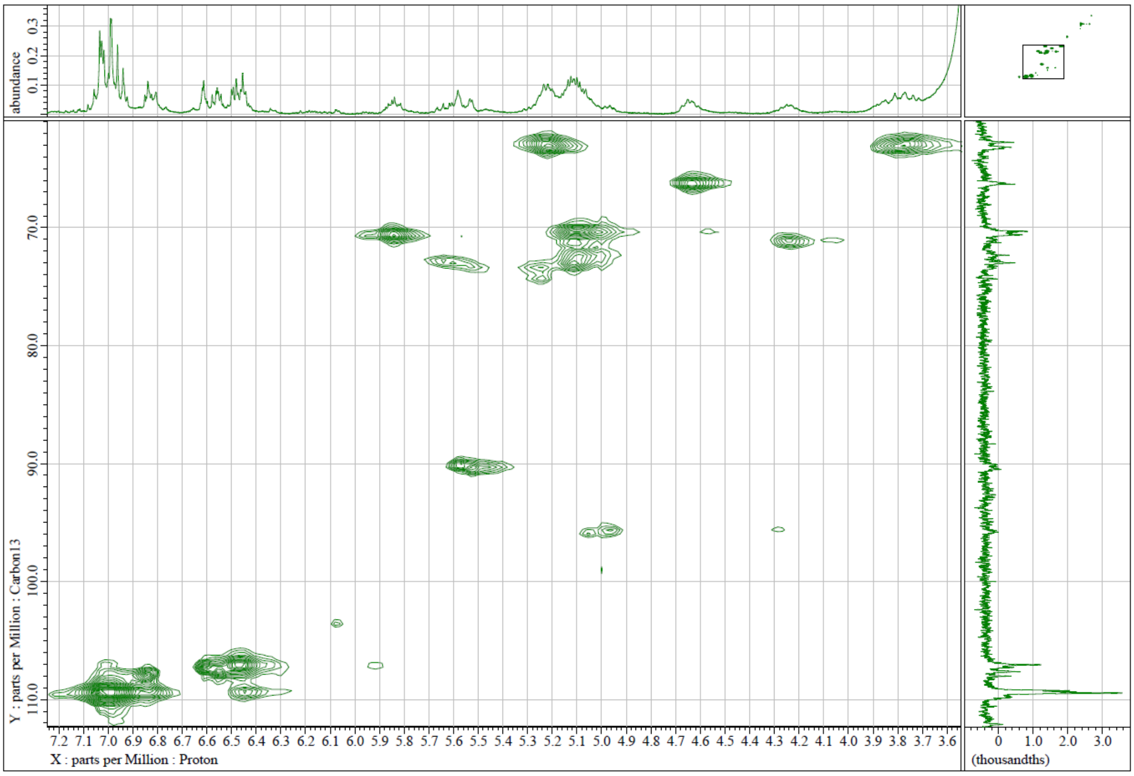

Figure S3D

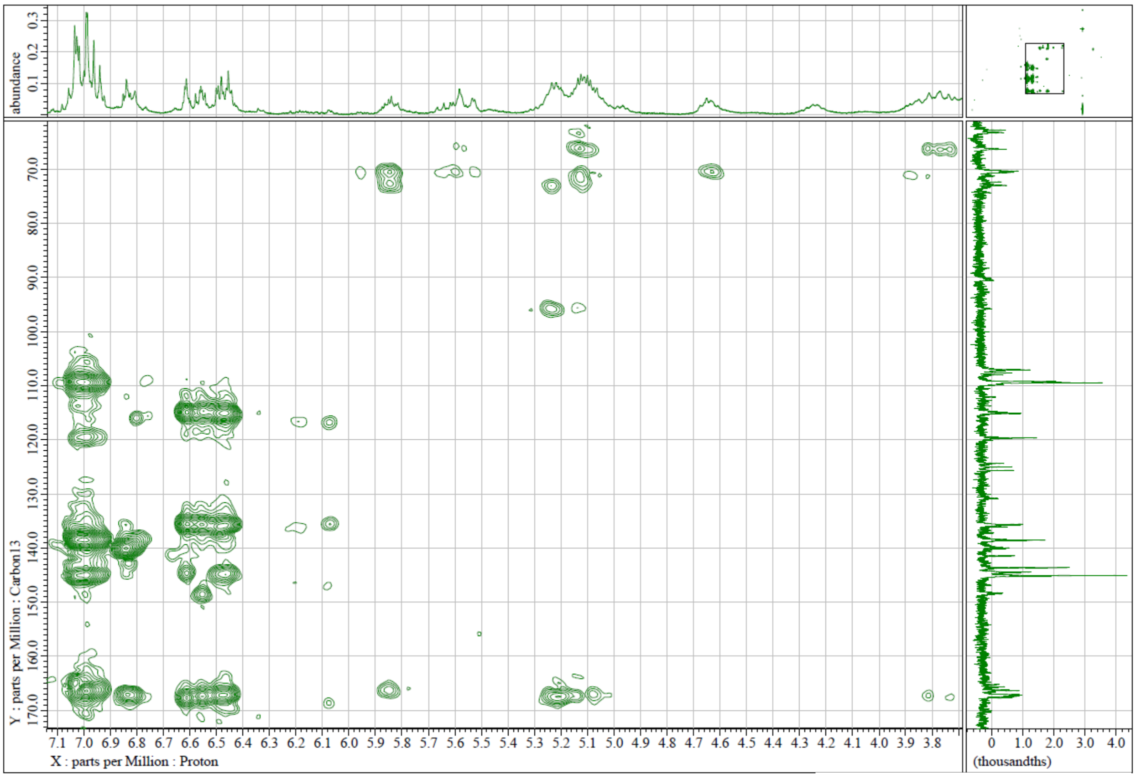
abundance
Y : parts per Million : Carbon13
X : parts per Million : Proton
(thousandths)

Figure S3E

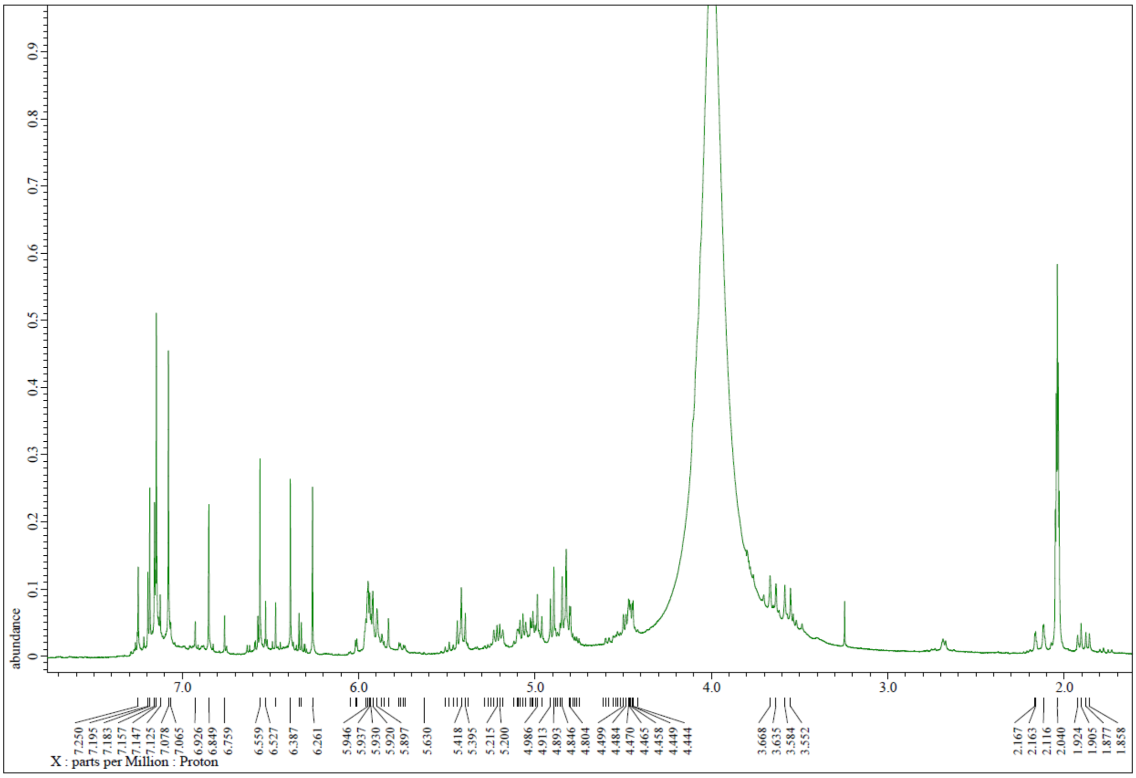

Figure S4A

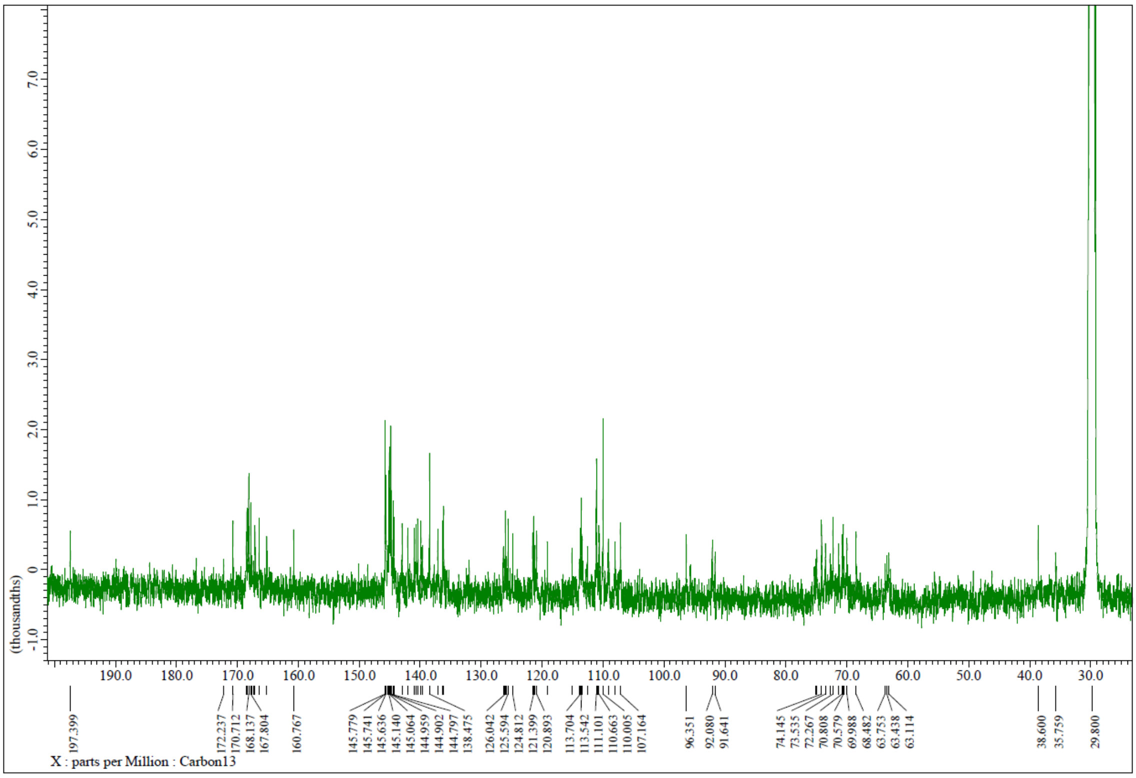

Figure S4B

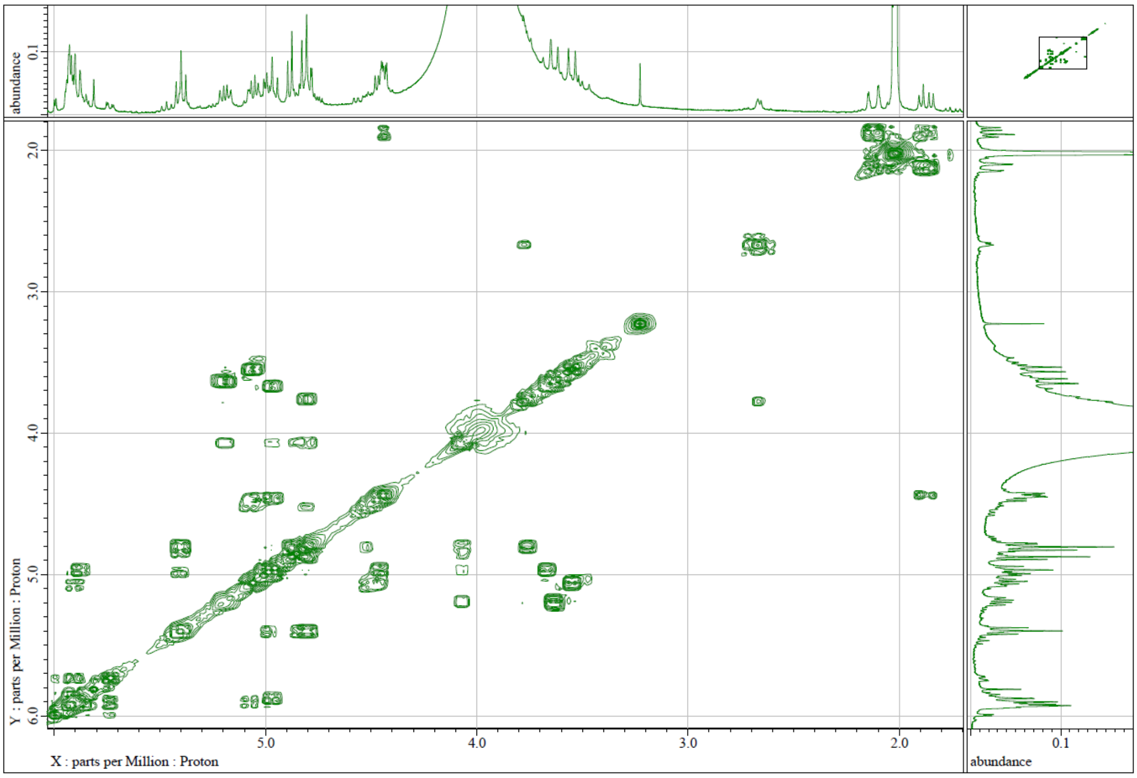

Figure S4C

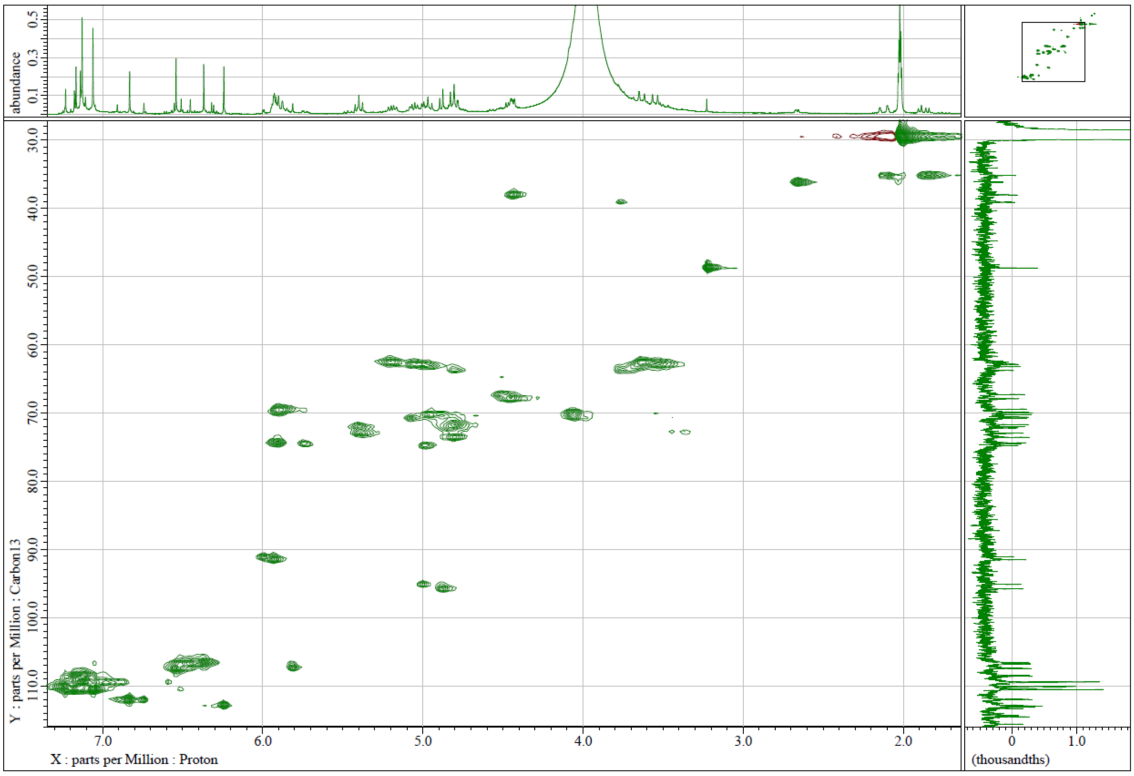

Figure S4D

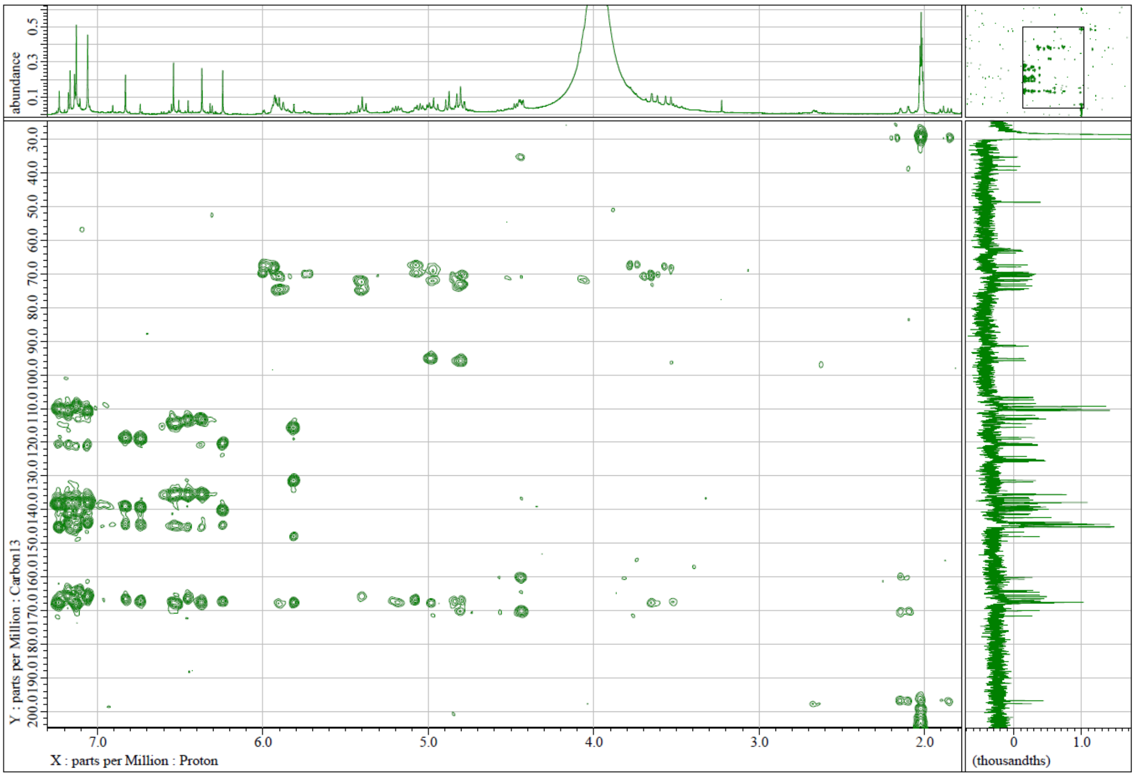
abundance
X : parts per Million : Proton
Y : parts per Million : Carbon13
(thousandths)

Figure S4E
